# Network structure influences self-organized criticality in neural networks with dynamical synapses

**DOI:** 10.3389/fnsys.2025.1590743

**Published:** 2025-06-18

**Authors:** Yoshiki A. Sugimoto, Hiroshi Yadohisa, Masato S. Abe

**Affiliations:** ^1^Graduate School of Culture and Information Science, Doshisha University, Kyotanabe, Kyoto, Japan; ^2^Faculty of Culture and Information Science, Doshisha University, Kyotanabe, Kyoto, Japan; ^3^Center for Advanced Intelligence Project, RIKEN, Chuo-ku, Tokyo, Japan; ^4^CBS-TOYOTA Collaboration Center, RIKEN, Wako, Saitama, Japan

**Keywords:** brain criticality hypothesis, self-organized criticality (SOC), neuronal avalanches, Dragon king, network structure, synaptic plasticity, neural dynamics

## Abstract

The brain criticality hypothesis has been a central research topic in theoretical neuroscience for two decades. This hypothesis suggests that the brain operates near the critical point at the boundary between order and disorder, where it acquires its information-processing capabilities. The mechanism that maintains this critical state has been proposed as a feedback system known as self-organized criticality (SOC); brain parameters, such as synaptic plasticity, are regulated internally without external adjustment. Therefore, clarifying how SOC occurs may provide insights into the mechanisms that maintain brain function and cause brain disorders. From the standpoint of neural network structures, the topology of neural circuits also plays a crucial role in information processing, with healthy neural networks exhibiting small world, scale-free, and modular characteristics. However, how these network structures affect SOC remains poorly understood. In this study, we conducted numerical simulations using a simplified neural network model to investigate how network structure may influence SOC. Our results reveal that the time scales at which synaptic plasticity operates to achieve a critical state differ depending on the network structure. Additionally, we observed Dragon king phenomena associated with abnormal neural activity, depending on the network structure and synaptic plasticity time scales. Notably, Dragon king was observed over a wide range of synaptic plasticity time scales in scale-free networks with high-degree hub nodes. These findings highlight the potential importance of neural network topology in shaping SOC dynamics in simplified models of neural systems.

## 1 Introduction

A number of empirical and theoretical studies have examined the brain criticality hypothesis, which is derived from an idea in statistical physics and complex systems science (Beggs and Timme, 2012; O'Byrne and Jerbi, 2022). In brief, this hypothesis posits that neural networks operate near a critical point that is the boundary between disordered and ordered phases, and exhibit critical phenomena whose sizes follow a power law distribution and long-range spatial or temporal correlations. Also, the properties stemming from criticality can maximize some functions such as information processing capabilities and transmissions, suggesting that a brain benefits from being critical (Shew and Plenz, 2013; Bertschinger and Natschläger, 2004; Kinouchi and Copelli, 2006; Boedecker et al., 2012; Avramiea et al., 2022; Haldeman and Beggs, 2005; Safavi et al., 2024). While much empirical evidence that brains exhibit critical properties has been accumulated (Petermann et al., 2009; Shew et al., 2009; Friedman et al., 2012; Tagliazucchi et al., 2012; Shriki et al., 2013), a considerable number of studies reported that brain disorders are associated with non-critical dynamics [reviewed in Zimmern (2020)]. For example, in the brain dynamics of individuals with neurological disorders such as epilepsy and schizophrenia, there was a deviation from the power law neural avalanche (Meisel et al., 2012; Poil et al., 2012). Considering the benefits of criticality, it is natural that brain disorders may be influenced by loss of criticality. Thus, it is essential to elucidate how the deviations from critical states occur for understanding, predicting brain disorders.

For the brain to be critical, the factors (i.e., control parameters) underlying the brain system, such as synaptic strength, must be at or near the appropriate values, i.e., critical value. How are such parameters of the neural networks of (healthy) individuals tuned to be the value? It has been suggested that self-organized criticality (SOC) plays an important role in that (Beggs and Plenz, 2003; Levina et al., 2007; Meisel and Gross, 2009; Rubinov et al., 2011; Ma et al., 2019). SOC refers to the property of feedback systems that are homeostatically keeping a critical state based on internal rules without external controls. An example of SOC in the brain is synaptic plasticity by which synaptic strength is adaptively tuned to a desirable state (Hesse and Gross, 2014; Beggs and Plenz, 2003; Ma et al., 2019). Previous studies showed that control parameters determining the system dynamics can hover around a critical point depending on the parameters of the plasticity rules or homeostatic mechanisms that give rise to SOC (Bonachela and Munoz, 2009; Bonachela et al., 2010; Kinouchi et al., 2019). This results in not a critical system perfectly tuned but a quasi-critical system.

Moreover, a peculiar phenomenon known as “Dragon king” has been identified in complex systems through SOC (self-organized criticality) (Sachs et al., 2012; Kinouchi et al., 2019). Dragon kings refer to events that appear as statistically significant outliers within distributions that otherwise follow a power law, characterized by their extremely large scale and departure from the overall scaling behavior (Sornette, 2009). Such phenomena have been observed in a variety of systems, including financial markets, earthquakes, weather systems, and the brain (Sornette and Ouillon, 2012; Lei, 2012; Peters et al., 2012; Süveges and Davison, 2012; Osorio et al., 2010), and in the brain, they are thought to be associated with dysfunctions in neural activity, such as epilepsy. Unlike typical large events observed in supercritical states, Dragon kings are distinguished by their qualitative and statistical singularity. That is, they are extreme outliers that do not follow the expected scaling laws, such as power law distributions. Importantly, these events are not merely large-scale events that become more frequent in the supercritical regime, but are instead associated with distinct internal feedback mechanisms that destabilize the equilibrium maintained by SOC (Kinouchi et al., 2019). In neuroscience, this distinction is of particular importance. This is because Dragon king-like events—such as pathological epileptic seizures—are not simply exaggerated versions of typical neural avalanches, but are believed to emerge from the breakdown of regulatory systems (e.g., synaptic plasticity) that normally serve to stabilize criticality. Thus, the occurrence of Dragon kings may indicate a collapse of SOC itself. Such abnormal neural activities are thought to arise from a failure in the self-organizing mechanisms that sustain criticality. For example, numerical simulations of spiking neural networks have reported that abrupt fluctuations in synaptic strength can trigger Dragon king-like events in the distribution of neuronal firing (Kinouchi et al., 2019). Furthermore, an analytical solution of a simplified model has demonstrated that the interplay between the increase and decrease of a control parameter determines whether Dragon kings emerge (Mikaberidze et al., 2023). These findings suggest that the parameters governing SOC can have a direct impact on the dynamics and functional performance of the neural system. Therefore, understanding the emergence of Dragon kings is not just merely a refinement of criticality theory, but is also crucial for identifying and potentially preventing pathological brain states that result from failed self-organization. This makes the study of Dragon king phenomena highly relevant to both theoretical neuroscience and clinical applications.

In general, brain dynamics underlies network structures (i.e., topology). While experimental evidence suggests that healthy neural networks possess three key network structures: small worldness, modularity, and scale-freeness (Stam, 2004; Achard et al., 2006; Bassett et al., 2010), in neural networks associated with certain health issues, one or more of these properties are often found to be lacking (Heiney et al., 2021). Although these facts indicate that network structures have important roles in information processing, how properties of networks contribute to brain functions related to information processing remains insufficiently explored. One possible direction is to explore the relationship between brain criticality and network structures. Previous studies show how the critical point changes or the critical regime stretches depending on the structure of the neural network (Pazzini et al., 2021; Kinouchi et al., 2019; Costa et al., 2017; Wang and Zhou, 2012; Moretti and Muñoz, 2013). However, the way in which network structure affects the characteristics of SOC is not fully understood.

In this study, we numerically investigated how structural characteristics of simplified neural networks may contribute to realizing and maintaining a critical state, within the framework of SOC. By constructing various network structures, ranging from random networks to small world, modular, and scale-free networks, we examined how each structure may affect the emergence of power law distributed neural avalanches and the occurrence of Dragon king phenomena. Particular attention was given to the interplay between synaptic plasticity timescales and network topology. The findings of this modeling study are expected to provide insights into the potential relationships between network structure and SOC-related dynamics, shedding light on missing links in the understanding of SOC mechanisms.

## 2 Methods

### 2.1 Modeling of neural networks

To model neural networks, we utilize the discrete time stochastic Leaky Integrate-and-Fire (LIF) model following previous studies (Kinouchi et al., 2019; Pazzini et al., 2021). This model expresses the phenomenon of neuronal firing, in which a neuron generates an electrical signal when the membrane potential exceeds a certain threshold and transmits it to other connected neurons. In a deterministic LIF model, the same input always generates the same output. However, in the actual brain (for example, in cortical neurons), although generally stable responses are observed, slight variability can be seen across trials, and even when the same stimulus is presented repeatedly, some variation in the neuronal response is confirmed (Bryant and Segundo, 1976; Mainen and Sejnowski, 1995). Therefore, in this study, we use a stochastic LIF model to account for the subtle stochastic fluctuations.

Let *N* denote the number of neurons, and the firing of neuron *i* at time *t* is represented by *X*_*i*_[*t*], where *X*_*i*_ = 1 indicates firing and *X*_*i*_ = 0 indicates non-firing. Let *V*_*i*_[*t*](≥ 0) represent the membrane potential of neuron *i* at time *t*. If neuron *i* fires at time *t*, then its membrane potential at time *t* + 1 is set to *V*_*i*_[*t* + 1] = 0.

When there is a connection from neuron *j* to neuron *i*, let neuron *j* be the presynaptic cell and neuron *i* the postsynaptic cell. The synaptic strength from the neuron *j* to *i* is denoted as *W*_*ij*_(> 0), and the reverse value *W*_*ji*_ is set as 0 because of unidirectionality. If there is no connection between neurons *i* and *j*, both *W*_*ij*_ and *W*_*ji*_ are set to 0. The time evolution of the membrane potential *V*_*i*_ is following:


(1) $$\begin{array}{l} V_{i}\left[t+1\right]=\mu_{i}V_{i}\left[t\right]+I_{i}\left[t\right]+\frac{1}{k_{i}}\overset{N}{\underset{j=1}{\sum }}W_{ij}X_{j}\left[t\right]. \end{array}$$

Here, μ_*i*_ and *I*_*i*_[*t*] represent the leak parameter and external input at time *t*, respectively. Then, *k*_*i*_ is the number of inputs to the neuron *i*.

The firing probability of the neuron *i* at time *t* is determined by its membrane potential *V*_*i*_[*t*]. Thus, let *P*(*X*_*i*_[*t*] = 1|*V*_*i*_[*t*]) denote it. We simply assume that the probability becomes non-zero when *V*_*i*_ exceeds the threshold θ_*i*_, and the larger the value of *V*_*i*_, the higher the firing probability. On the other hand, when the membrane potential *V*_*i*_ does not exceed the threshold θ_*i*_, neuron *i* may still fire spontaneously with a small probability, denoted as *p*_spont_. This relationship is expressed as follows:


(2) $$\begin{array}{l} P\left(X_{i}[t]=1|V_{i}[t]\right)\equiv \{\begin{array}{ll} \text{Φ}\left(V_{i}[t]\right) & \text{ if }V_{i}[t]-\theta_{i}>0,\\ p_{\text{spont }} & \text{ otherwise, } \end{array} \end{array}$$

(3) $$\begin{array}{ll} \Phi \left(V_{i}\left[t\right]\right) & =\frac{\Gamma_{i}\left(V_{i}\left[t\right]-\theta_{i}\right)}{1+\Gamma_{i}\left(V_{i}\left[t\right]-\theta_{i}\right)}. \end{array}$$

In Equation 3, Γ_*i*_ represents the gain parameter of the neuron *i* (with Γ_*i*_ > 0.0). Consequently, when the membrane potential *V*_*i*_ of the neuron *i* exceeds the threshold value θ_*i*_ (*V*_*i*_ > θ_*i*_), the firing probability of the neuron *i* is determined by an increasing function of the gain parameter Γ_*i*_ and the difference between the membrane potential *V*_*i*_ and the threshold θ_*i*_. In this study, unless otherwise specified, the following parameters are used: *N* = 10, 000, μ_*i*_ = 0.0, *I*_*i*_[*t*] = 0.0, *p*_spont_ = 0.0001, Γ_*i*_ = 0.8, and θ_*i*_ = 0.0.

### 2.2 Derivation of the critical value

To estimate the critical value *W*_*c*_ of the average synaptic strength, we calculate the susceptibility χ_*s*_. The avalanche size *s* is defined as the total number of neurons that become active, from the moment when the number of spikes first becomes non-zero among the 400 neurons to the point when it returns to zero. Susceptibility is a well-established metric that quantifies the sensitivity or instability of the dynamics of a system, even in the absence of external input, and is theoretically known to exhibit a peak at the critical point (Fisher, 1967; Stanley, 1987). Consequently, the critical point can be inferred as the value of *W* at which χ_*s*_ reaches its maximum. In this analysis, we focus on the case in which the average synaptic strength *W* remains constant over time. The simulations were carried out by varying *W* from 0.00 to 4.00 in increments of 0.01. For each value of *W*, we performed sufficiently long simulations, recorded all avalanche sizes *s*, and computed the mean 〈*s*〉 and the second moment 〈*s*^2^〉. The susceptibility χ_*s*_ was then defined as:


(4) $$\begin{array}{l} \chi_{s}=\langle s^{2}\rangle -{\langle s\rangle }^{2} \end{array}$$

By plotting χ_*s*_(*W*), we identified the value of *W* that maximized the susceptibility, and defined this value as the critical synaptic strength *W*_*c*_. The result is shown in Supplementary Figure S1.

### 2.3 Mathematical model for self-organized criticality

For SOC in neural network models, various mathematical models have been proposed in previous studies (Kinouchi et al., 2020). Among these, we consider a model in which the synaptic strength *W*_*ij*_ fluctuates over time according to increasing and decreasing only when neuron *j* is connected to neuron *i* following a previous study (Pazzini et al., 2021). The time evolution of *W*_*ij*_ is described by


(5) $$\begin{array}{l} W_{ij}\left[t+1\right]=W_{ij}\left[t\right]+\frac{1}{\tau }-uW_{ij}\left[t\right]X_{j}\left[t\right]. \end{array}$$

Here, τ(> 0) is the time constant that determines how slow the synaptic strength *W*_*ij*_ increases, and *u*(0 < *u* < 1) is the synaptic depression coefficient. In summary, the synaptic strength *W*_*ij*_ for the connection from presynaptic neuron *j* to postsynaptic neuron *i* increases by a fixed amount at each time step but decreases when the presynaptic neuron *j* fires. This mathematical model assumes synaptic plasticity and provides a simplified model for potentially reproducing SOC. The initial value of the synaptic strength *W*_*ij*_[0] is set to the critical value *W*_*c*_, and numerical analysis is performed.

### 2.4 Deviation index from the critical value of synaptic strength

As an indicator of how much the average synaptic strength *W*[*t*] of the entire network at time *t* deviates from the critical value *W*_*c*_, we calculate the Mean of Error (ME) and Mean of Absolute Error (MAE) following:


(6) $$\begin{array}{l} ME=\frac{1}{t_{b}-t_{a}+1}\overset{t_{b}}{\underset{t=t_{a}}{\sum }}\left(W\left[t\right]-W_{c}\right), \end{array}$$

(7) $$\begin{array}{l} MAE=\frac{1}{t_{b}-t_{a}+1}\overset{t_{b}}{\underset{t=t_{a}}{\sum }}|W\left[t\right]-W_{c}|. \end{array}$$

Specifically, we compute the average of the difference between *W*[*t*] and the critical value *W*_*c*_ over the period from $t_{a}=10^{4}$ to $t_{b}=10^{5}$. In this way, we analyze the variation in synaptic strength across the entire network. However, for neurons with an in-degree of 0 or 1, the synaptic strength *W*_*ij*_ follows Equation 5 and continuously increases by 1/τ at each time step. This is because the decrease term in the plasticity rule, which depends on the activity of the presynaptic neuron *j*, only takes effect when that neuron fires. In the case of in-degree 0, there is no presynaptic input at all, so the synaptic strength keeps increasing without constraint. Even for in-degree 1, if the single presynaptic neuron rarely fires, the decrease term is seldom applied, leading again to unbalanced growth of *W*_*ij*_. As a result, these neurons can influence the accurate evaluation of the network-wide mean synaptic strength *W*[*t*] at time *t*. Therefore, when calculating the mean synaptic strength *W*[*t*] for the entire network, the synaptic strengths *W*_*ij*_ of these neurons are excluded from consideration.

### 2.5 Construction of neural networks

To construct a directed network that has small world properties, scale-free characteristics, or modular structures, we use the Watts-Strogatz (WS) model (Watts and Strogatz, 1998), the Barabási-Albert (BA) model (Barabási and Albert, 1999), and the Stochastic Block (SB) model (Holland et al., 1983). Based on these models, we generate five types of network structures: a regular network, a small world network, a scale-free network, a modular network, and a random network constructed from the Erdős-Rényi (ER) model (Erdős and Rényi, 1959), with the number of neurons and synaptic connections kept consistent. The direction of links is randomly determined. To avoid immediate recurrent loops that could obscure causal relationships in cascade dynamics, bidirectional connections are excluded. This modeling choice allows us to isolate the effects of network structure and synaptic plasticity without interference from short feedback cycles. Details on the construction of the modular network are provided in the Supplementary Section 1.1. Unless otherwise specified, we construct networks with an average degree of eight in this study. The clustering coefficients of the networks used in this study are provided in the Supplementary Table 1.

### 2.6 Classification of neural network dynamics

To understand the synaptic plasticity conditions under which neuronal avalanches occur, we classify the state of the neural network using the firing distribution based on the total number of neurons that fired in a cascade. When the neural network reaches a critical state, cascades of varying sizes occur, and the distribution follows a power law. On the other hand, when large-scale neuronal firings occur frequently, resulting in a deviation above the power law, a “Dragon king” phenomenon can occur (Sornette, 2009). One method to determine whether the Dragon king phenomenon is present is to test whether the distribution statistically matches a power law (Janczura and Weron, 2012; Pisarenko et al., 2014). Furthermore, studies investigating how Dragon kings arise in SOC have also employed statistical hypothesis testing (Mikaberidze et al., 2023). However, in studies such as this one, which considers neural networks, the distribution may not always follow a typical power law but a truncated power law. Since no methods have been proposed to address such cases, we do not adopt the statistical hypothesis testing methods used in previous studies. Therefore, in this study, to understand the synaptic plasticity conditions under which neuronal avalanches occur, we classify firing states based on the distribution of the total number of neurons involved in cascading firings into four categories: “Dragon king” (deviations from the power law due to frequent large-scale cascading firings), “Subcritical” (insufficient firing), “Critical” (a critical state following the power law), and “Supercritical” (always large-scale cascading firings). Let the total number of neurons that fire in a cascade (i.e., the avalanche size) be denoted as *s*. The vector of avalanche sizes generated by simulation is represented as **s**. After performing numerical analyses up to *t* = 100, 000 over a sufficient period, the values of two parameters, τ, and *u*, in Equation 5, which determine the timescale of synaptic plasticity, may lead to prolonged cascades of neuronal firing. As a result, the size of **s** may become small. In this study, if the number of elements in vector **s** is five or fewer (i.e., |**s**| ≤ 5), the state is classified as supercritical. For cases where this condition is not met, the remaining states are classified into three categories. To avoid dependence on the initial state, the first five elements of **s** [i.e., (*s*_1_, *s*_2_, *s*_3_, *s*_4_, *s*_5_)] are removed before constructing the Complementary Cumulative Distribution Function (CCDF). When the total number of neurons in cascades is small [in this study, if the maximum value of elements in **s** is 10^2.5^ or less, i.e., max(**s**) ≤ 10^2.5^], the state is classified as subcritical. For cases with sufficiently large cascade sizes, states are classified as either dragon king or critical. This classification is determined by fitting the CCDF to a truncated power law and evaluating whether the observed distribution deviates from the fit. For details on the method of fitting the CCDF to a truncated power law, please refer to the Supplementary Section 1.3. The use of a truncated power law instead of a simple power law is justified by the finite size of the network, which can introduce cutoffs in the power law behavior. Specifically, when CCDF(10^2^) = *P*(*s*≥10^2^) < 10^−2^ and the CCDF deviates by more than 1.1 times from the CCDF predicted by the truncated power law, the state is classified as dragon king. Supplementary Figure S2 presents the CCDF classified into these four categories using this method.

## 3 Results

**Figure 1:**
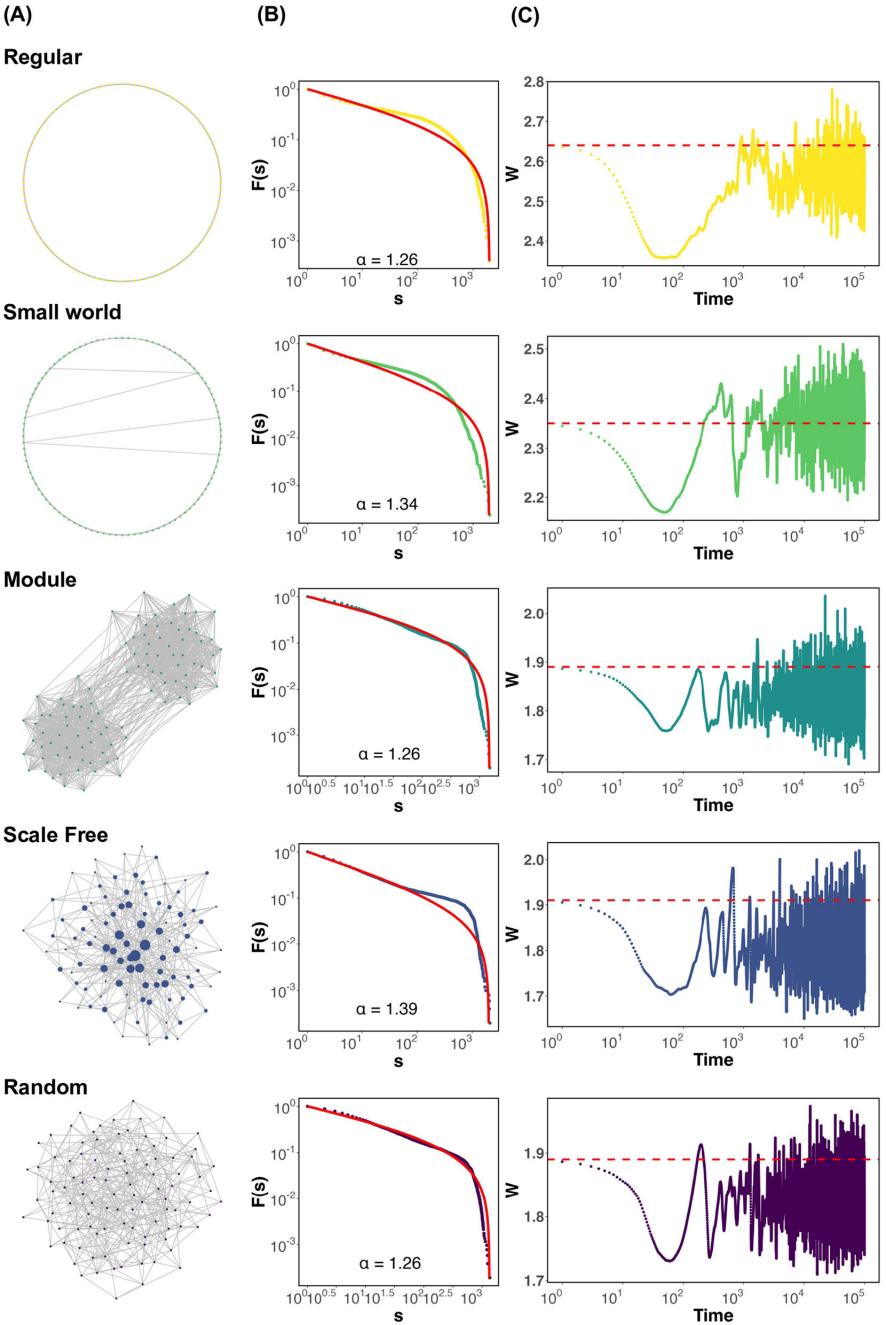
Firing distribution of neurons and time evolution of the average synaptic strength *W* for each network structure, assuming synaptic plasticity with τ = 500 and *u* = 0.1. **(A)** Visualization of networks. All networks are drawn with *N* = 100 nodes and *m* = 400 edges for display purposes. In the scale-free network, the nodes are drawn larger in proportion to their degree. **(B)** CCDF based on the total number of neurons that have been fired in a chain reaction, *s*. The red solid line represents the result of fitting the distribution to a truncated power law. α is the power law exponent. **(C)** Visualization of the time evolution of the average synaptic strength *W*. The red dashed line represents the critical value *W*_*c*_.

First, we present graphs showing the state of each neural network (Figure 1A), the conditions under which criticality occurs, and the deviation of the average synaptic strength *W* from the critical value *W*_*c*_ when the parameters of Equation 5 are set to τ = 500 and *u* = 0.1 as the timescale. These include the cascade size distribution (Figure 1B), the time-series graph of the average synaptic strength *W*[*t*] (Figure 1C). The probability density function (PDF) corresponding to Figure 1B is provided in the Supplementary Figure S3. The firing distribution adheres to a truncated power law in modular and random networks (Figure 1B). In the regular, small world, and scale free networks, dragon king events—departures from the power law distribution—are observed (Figure 1B; Regular, Small world, Scale free). Here, it is important to note that power law behavior is not unique to the critical state. To confirm the scaling laws involving multiple power laws near the critical point *W*_*c*_, we analyzed the relationship between the number of neurons firing in cascades, the duration of firing events, and the interplay between these two factors (Sethna et al., 2001; Scarpetta et al., 2018). These results are provided in the Supplementary Figure S10. The findings suggest that, across all network structures, the relationship between the number of neurons firing in cascades and the duration of firing events approximately follows a scaling trend. However, noticeable deviations from the theoretical slope (α−1)/(β−1) were observed. This mismatch may reflect the fact that strict assumptions required for scaling laws—such as temporal scale separation and stationarity—are not necessarily satisfied in systems exhibiting self-organized criticality. Indeed, in regular, small world, and scale-free networks, we observed Dragon king events, which represent large deviations from power law behavior (Figure 1B). These deviations suggest that the system may depart from criticality, or that multiple dynamical regimes coexist. Therefore, while the observed data display features consistent with critical-like behavior, they should not be interpreted as definitive evidence of criticality. Further investigation is needed to develop robust criteria for identifying criticality in self-organized neural systems. Additionally, while the average synaptic strength *W* in all networks demonstrates SOC behavior, variations in *W* depend on the network structure (Figure 1C). Specifically, *W* fluctuates below the critical value *W*_*c*_ in networks with high clustering coefficients, such as regular and small world networks (Figure 1C; Regular, Small world). Conversely, in modular, scale free, and random networks, *W* fluctuates near *W*_*c*_ (Figure 1C; Module, Scale free, Random). These findings demonstrate that, under the assumption of synaptic plasticity with a timescale of τ = 500 and *u* = 0.1, dragon king events occur in regular, small world, and scale free networks. Moreover, regardless of this outcome, variations in the behavior of the average synaptic strength *W* depend on the network structure. Supplementary Figure S4 shows the trajectories of average synaptic strength *W* and average firing rate ρ for each network. The method for calculating the average firing rate ρ of neurons, which will appear frequently in the following sections, is provided in the Supplementary Section 1.4. In this study, the initial value for the simulations was set to the critical value *W*_*c*_. However, even when the initial value was set to a different value away from the critical point, it was confirmed that the system ultimately converged to *W*_*c*_, indicating that the conclusions of this study are not significantly affected by the choice of initial conditions (Supplementary Figure S5). A previous study also reported this point (Pazzini et al., 2021). The emergence of criticality may partly depend on the specific form of the synaptic plasticity rule (Supplementary Figure S6). To further explore the role of network geometry, we conducted additional simulations using two-dimensional (2D) and three-dimensional (3D) regular lattice networks, each configured to match the node number and degree of the one-dimensional (1D) regular network. The results showed that, in both 2D and 3D networks, the average synaptic strength *W* remained close to the critical value *W*_*c*_, with no significant deviation. This contrasts with the deviation observed in the 1D regular network and suggests a dimensional dependence in the emergence and stability of self-organized criticality. Detailed results are provided in the Supplementary Figure S9. To further investigate the influence of network structure, we conducted simulations using the Watts–Strogatz model with various rewiring probabilities *p* (*p* = 0.0001, 0.001, 0.1), thereby generating networks with different degrees of small world characteristics. This allowed us to examine a continuum of networks ranging from regular to random structures. The results of these simulations are presented in the Supplementary Figure S9.

**Figure 2:**
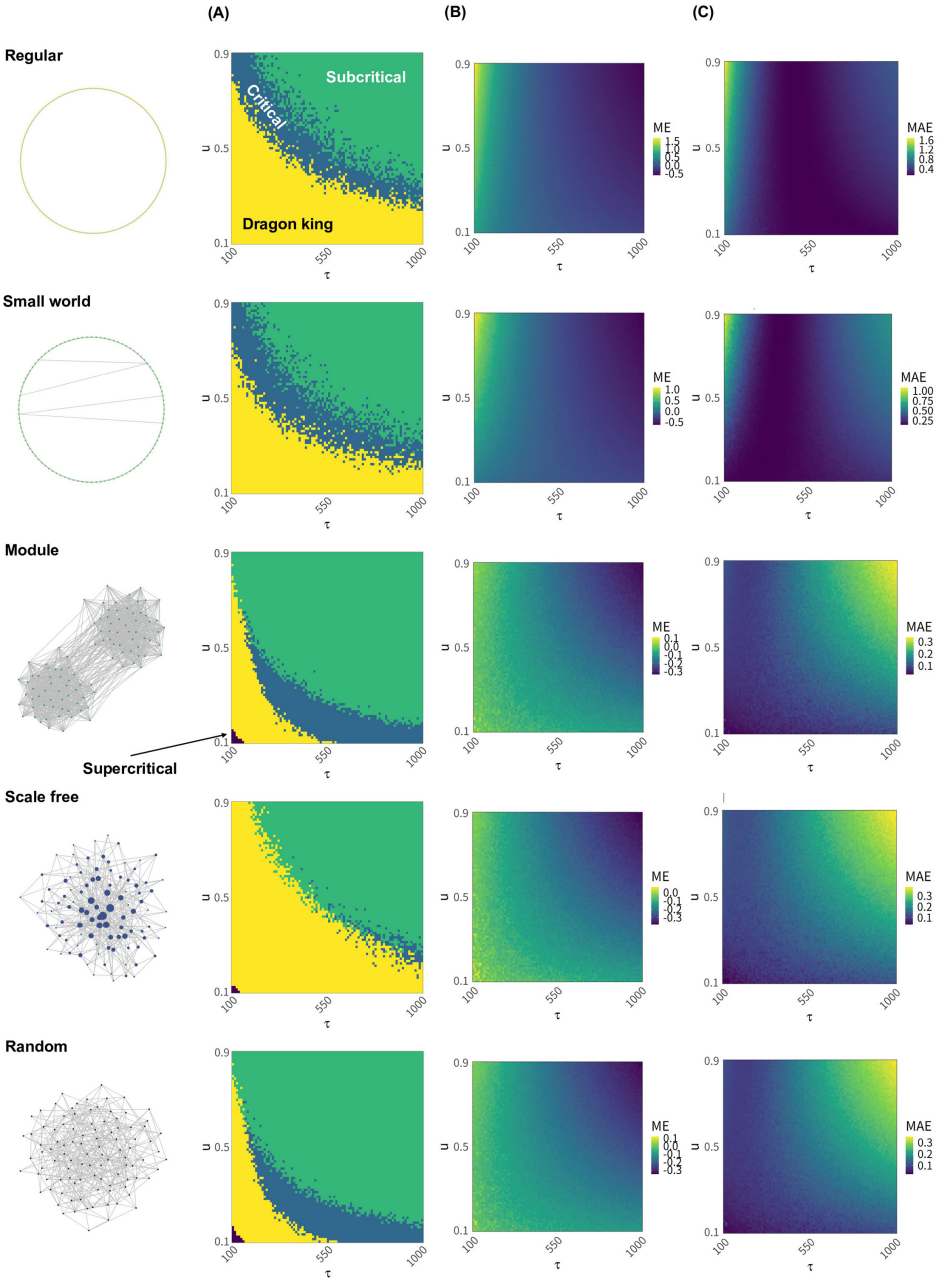
The state of the neural network and the deviation of the average synaptic strength *W* from the critical value *W*_*c*_, assuming various synaptic plasticity conditions and different neural network structures. The state of the neural network and the deviation of the average synaptic strength *W* from the critical value *W*_*c*_ were compared across different network structures when various synaptic plasticity conditions were assumed within the range of 100 ≤ τ ≤ 1000 and 0.1 ≤ *u* ≤ 0.9 in Equation 5. **(A)** For each value of the time constant τ and synaptic depression coefficient *u*, the state of the neural network is classified as “Dragon king,” “Subcritical,” “Critical,” or “Supercritical,” and the results are indicated by color. **(B)** For each value of the time constant τ and synaptic depression coefficient *u*, the degree of deviation of the average synaptic strength *W* from the critical value *W*_*c*_ is calculated using ME indicator according to Equation 6. **(C)** As in **(B)**, the deviation is computed for each τ and *u*, but using the MAE indicator defined in Equation 7.

Next, we present distribution of the neural avalanche of various neural networks under different timescales of synaptic plasticity and the deviations from the critical value *W*_*c*_ (Figure 2). Regular networks, small world networks, and scale-free networks exhibit a broader range of synaptic plasticity timescales where dragon king events occur compared to the other two network types (Figure 2A; Regular, Small world, Scale free). In particular, scale free networks rarely achieve a critical state across most synaptic plasticity timescales (Figure 2A; Scale free). For regular and small world networks, critical states are realized when the time constant τ is small, and the synaptic strength decay coefficient *u* is relatively large, or when τ is large and *u* is moderately small (Figure 2A; Regular, Small world). In contrast, modular networks and random networks achieve critical states when τ is large and *u* is small (Figure 2A; Module, Random). The extent to which the average synaptic strength *W* deviates from the critical value *W*_*c*_ also depends on the network structure, with regular and small world networks showing particularly pronounced deviations (Figure 2B; Regular, Small world). Moreover, independent of network structure, it was observed that the average synaptic strength *W* deviates from the critical value *W*_*c*_ depending on the value of τ (Figures 2B, C). Specifically, *W* tends to exceed *W*_*c*_ when τ is small, and fall below *W*_*c*_ when τ is large (Figure 2C). This deviation also varies with network structure and is particularly prominent in regular and small world networks (Figures 2B, C; Regular, Small world). In the main analysis, events were classified as Dragon kings when the CCDF deviated by more than 1.1 times from the cumulative frequency expected under a truncated power-law distribution. However, even when the classification threshold was changed to 1.3 or 1.5 times the expected frequency, the overall results remained largely unchanged (Supplementary Figures S14B, C). Additionally, changing the average degree of the network or the number of modules *B* in modular networks did not alter the timescales of synaptic plasticity required to achieve criticality (Supplementary Figures S12, S13). On the other hand, changes in the leak parameter of the LIF model significantly influenced the timescales of synaptic plasticity required to achieve criticality (Supplementary Figure S11). These findings suggest that the timescales of synaptic plasticity required to maintain a critical state vary depending on the structural properties of the network and the state of neural activity. Furthermore, they indicate that the correlation between the maintenance of criticality and the deviation of the mean synaptic strength from its critical value is influenced by the network structure (Supplementary Figure S7).

**Figure 3:**
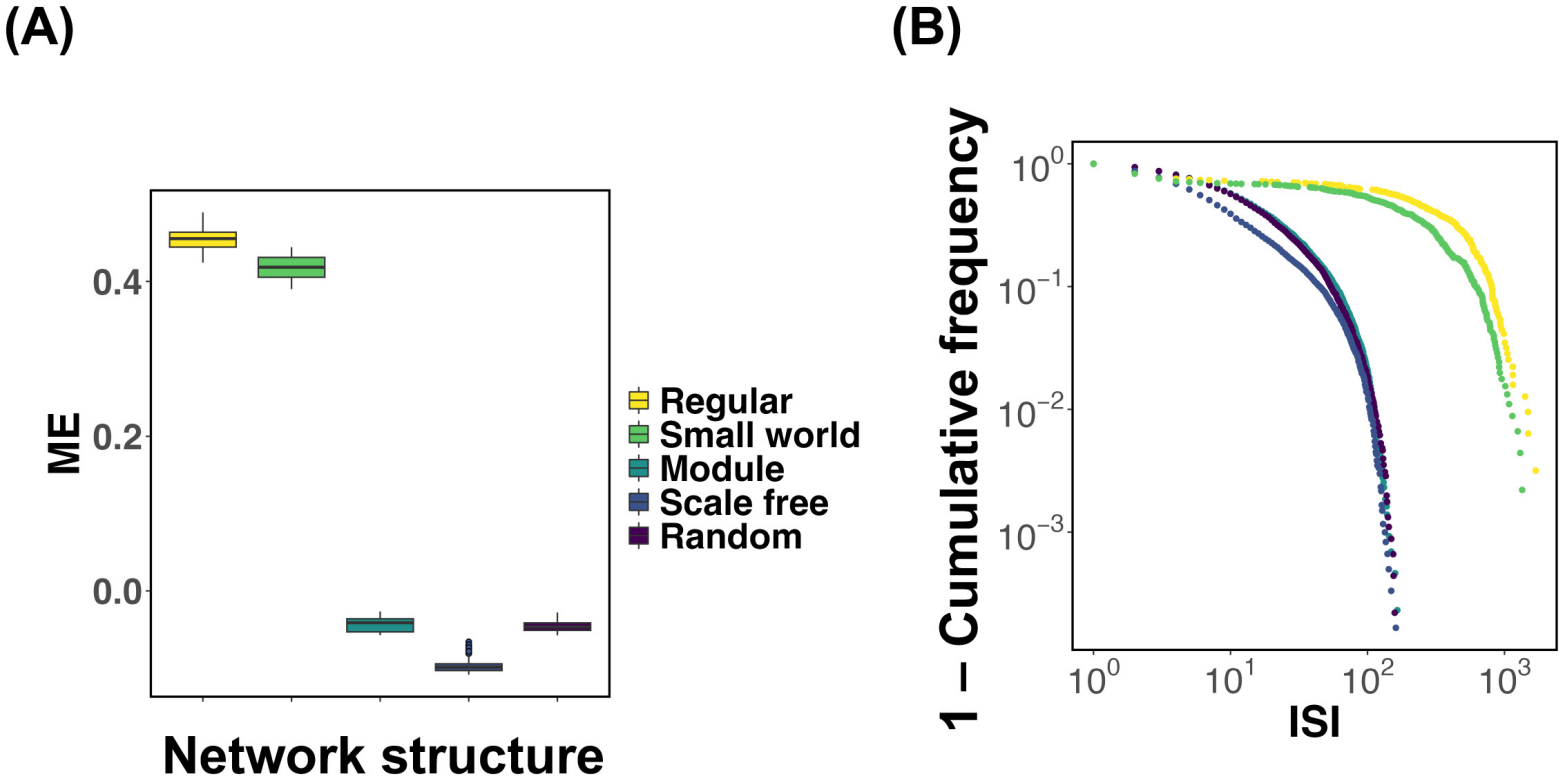
Comparison of the deviation of mean synaptic strength from the critical value *W*_*c*_ and inter-spike interval (ISI) distributions across different network structures. The numerical analysis conditions are τ = 200 and *u* = 0.5. **(A)** For each network structure, simulations were conducted 100 times, and the difference (ME) between the time-averaged synaptic strength during *t* = 10^4^~10^5^ and the critical value *W*_*c*_ was calculated. The box plots show the distribution of ME for each network structure. To evaluate the effect of network structure on ME, a one-way analysis of variance (ANOVA) followed by pairwise multiple comparisons was performed. Statistically significant differences were observed in all pairwise comparisons between network structures (*p* < 2.2 × 10^−16^). **(B)** Distributions of inter-spike intervals (ISI) for each network structure.

Based on the results obtained thus far, it has become evident that in regular and small world networks, the mean synaptic weight *W* deviates from the critical value *W*_*c*_ (Figures 2B, C; Regular, Small world). We investigated the extent to which the mean synaptic strength deviates from the critical value *W*_*c*_ for each network structure, focusing on a specific synaptic plasticity parameter. Figure 3A presents the results, where simulations were repeated 100 times for each network type. For each trial, the difference (ME) between the time-averaged synaptic strength over the interval *t* = 10^4^~10^5^ and the critical value *W*_*c*_ was computed and visualized using box plots. To evaluate the effect of network structure on ME, we conducted a one-way analysis of variance (ANOVA). The results revealed statistically significant differences in ME between all pairs of network structures (*p* < 2.2 × 10^−16^). Next, we sought to clarify why the distribution of synaptic weights differs depending on the network structure. As shown in Equation 5, changes in synaptic strength *W*_*ij*_ are highly dependent on neuronal spiking activity. To investigate how frequently neurons fire in each network structure, we calculated the distribution of inter-spike intervals (ISI) for each network (Figure 3B). These results indicate that, in both regular and small world networks, the ISI distributions exhibit burst-like characteristics compared to those in other network types. This implies that in networks with predominantly local connectivity, neuronal firing tends to occur in spatially and temporally clustered patterns. In other words, when a neuron fires, its neighboring neurons tend to fire in rapid succession, leading to temporally concentrated activity (i.e., bursts). Such burst dynamics cause the synaptic weights to decrease rapidly, resulting in a sustained deviation of the mean synaptic weight *W* from the critical value *W*_*c*_.

**Figure 4:**
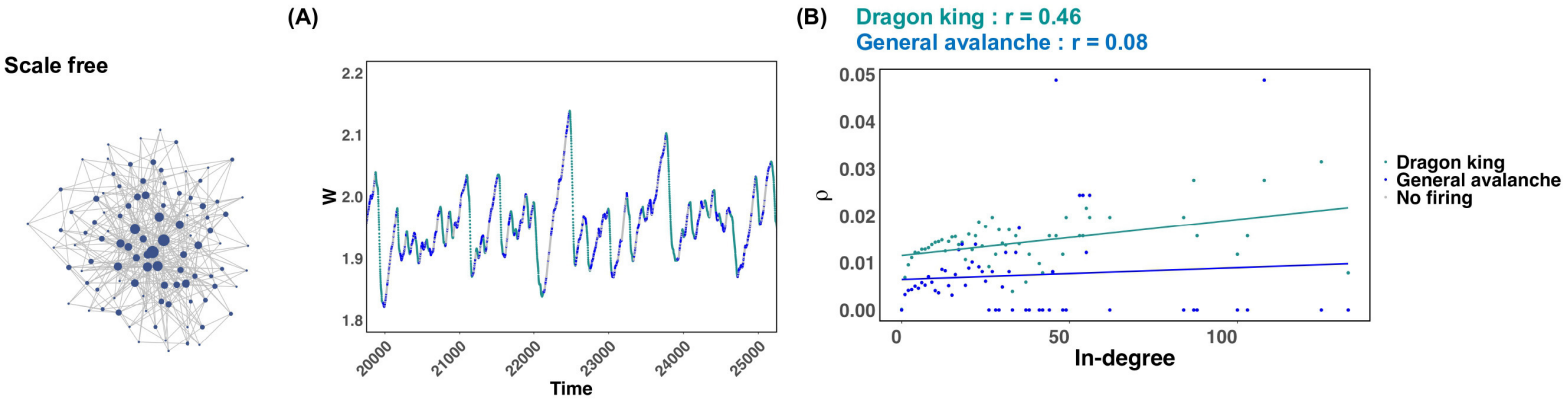
Behavior of the average synaptic strength *W* in scale free networks and the average firing frequency for both dragon king events and power law abiding neural avalanches. The numerical analysis conditions are τ = 600 and *u* = 0.1. **(A)** Behavior of the average synaptic strength *W* during the time interval *t* = 20, 000~25, 000. The states where dragon king events, power law abiding neural avalanches, and no firing occur are indicated by different colors. **(B)** The relationship between the in-degree of each neuron and the average firing rate ρ of the neurons. The two straight lines indicate regression lines.

In scale free networks, it was revealed that dragon king events occur under many synaptic plasticity timescales, and critical states are not realized (Figure 2B; Scale free). But why does this happen? To clarify this reason, we focused on a specific synaptic plasticity time scale in a scale-free network and plotted the average firing frequency ρ for each in-degree of neurons in both chain activations that followed a power law distribution and those identified as Dragon king events (Figure 4B). Figure 4A illustrates the timing of chain activations classified as following a power law distribution, those identified as Dragon king events, and periods of no activation, using color to indicate these events on the temporal evolution of the mean synaptic strength *W*. From the results shown in Figure 4A, it was evident that dragon king events occur during the periods when the average synaptic strength *W* decreases. This finding aligns with previous research (Mikaberidze et al., 2023). Furthermore, the results in Figure 4B reveal that in chain activations identified as Dragon king events, neurons with a higher in-degree (i.e., hub nodes) exhibit a higher average firing frequency ρ, indicating a positive correlation. In contrast, no such correlation is observed in chain activations that follow a power law distribution. These findings suggest that the presence of hub structures, characteristic of scale free networks, increases the average firing frequency of neurons. This leads hub nodes to fire more frequently than low-degree neurons, causing the neuronal firing distribution to deviate from the power law principle of “mostly small-scale firings with occasional large-scale events.” As a result, Dragon king events emerge.

## 4 Discussion

In this study, we numerically examined, using a mathematical model, the potential for neural network structures to induce critical states. First, it was revealed that the timing of functional synaptic plasticity required to achieve a critical state (commonly referred to as the time scale) differs depending on the network structure. Furthermore, depending on the network structure and the time scale of synaptic plasticity, the firing distribution of neurons was observed to deviate from the power law, resulting in the emergence of dragon kings. This phenomenon was particularly notable in scale-free networks containing high-degree hub nodes, where dragon kings were confirmed to occur over a wide range of synaptic plasticity time scales. These findings suggest that the interplay between the structural properties of neural networks and the time scale of synaptic plasticity may influence the realization and maintenance of critical states in simplified neural models. This result cannot be derived from mean-field approximations that disregard network structures or from SOC analyses using gain parameters as control variables in neurons (Kinouchi et al., 2019). Thus, this modeling study highlights, from the perspective of SOC, the potential importance of network topology in shaping critical dynamics.

Previous empirical studies have suggested that the presence of a “rich-club” structure in neural networks, where hubs are densely interconnected, facilitates efficient information transmission and functional integration across the entire brain (Van Den Heuvel and Sporns, 2011; Van den Heuvel and Sporns, 2013; Ball et al., 2014). In contrast to this perspective, we propose, based on a simplified neural network model, that in network structures where a small number of hubs connect to a majority of nodes, dynamic changes in synaptic strength based on the synaptic plasticity assumptions expressed by Equation 5 may hubs more likely to fire compared to low-degree neurons. Consequently, this affects the overall dynamics of the neural network, potentially resulting in abnormal neuronal firing phenomena such as Dragon king, potentially resulting in abnormal neuronal firing phenomena such as Dragon king. While previous studies highlighted the advantages of hubs, our modeling results suggest their potential to contribute to dynamic instabilities. Specifically, when hubs function normally, they enhance the efficiency of information transmission throughout the brain. However, if synaptic dynamics promote excessive firing of hubs, it could destabilize the dynamics of the network. Traditional studies have not sufficiently focused on the behavior of hubs within the SOC framework. However, our modeling study suggests that excessive firing of hubs could lead to significant changes in synaptic strength, affecting the overall network dynamics.

The structural characteristics of neural circuits play a crucial role in the regulation of synaptic strength and the maintenance of critical states. Our modeling results revealed that in networks with high clustering coefficients, the deviation of the average synaptic strength from its critical value was observed, in contrast to Random networks. Previous studies have indicated that the topology of neural networks influences neuronal firing patterns (Zhao et al., 2018; Nolte et al., 2020). Furthermore, in random networks, it has been confirmed that self-organized criticality is independent of the time scale of synaptic plasticity, ensuring that the system consistently operates near the critical value (Kinouchi et al., 2019). In contrast, in networks with high clustering coefficients, such as regular and small world networks, when a specific neuron fires, its adjacent neurons are highly likely to fire as well. This increase in local firing activity induces heterogeneity in the neural network, such as a reduction in synaptic strength in certain regions. Consequently, the average synaptic strength may deviate from the critical value. However, it was confirmed that these networks still retain the ability to maintain an overall critical state. Our results suggest that in simplified network models, networks with high clustering coefficients can tolerate local variations in synaptic strength and diversity in firing patterns while preserving criticality at the global level. This extension of conventional SOC frameworks observed in random networks may provide insights into the adaptability of more complex network structures. Additionally, this characteristic may reflect fundamental mechanisms that help balance stability and flexibility in neural circuits, potentially playing an important role in information processing.

Synaptic plasticity, which involves changes in synaptic strength, has a significant impact on the dynamics of neural networks and plays a crucial role in achieving and maintaining critical states (Stepp et al., 2015; Rubinov et al., 2011). When this plasticity functions appropriately, neural networks can perform efficient information processing and maintain stable functions. Previous studies have explained that short-term and long-term plasticity each plays distinct roles in achieving critical states (Zeraati et al., 2021). Short-term plasticity is considered to help maintain dynamics around the critical point by acting as a feedback mechanism through temporary changes in synaptic strength, keeping the system close to a critical state. Although this adjustment occurs rapidly, its effects are temporary and do not contribute directly to long-term stability. On the other hand, long-term plasticity is thought to play a role in stabilizing neural networks in critical states through sustained changes in synaptic strength. While this plasticity contributes to maintaining and stabilizing critical states, its direct relationship with learning and memory formation is not yet fully understood. Consequently, our modeling results suggest that achieving and maintaining critical states in simplified networks may not be directly linked to learning and memory formation, and these functions may need to be regarded as distinct processes. In this study, short-term plasticity corresponds to the upper-left regions of the plots in Figure 2, while long-term plasticity corresponds to the lower-right regions. In random networks and modular networks, critical states are achieved through long-term plasticity, consistent with previous research. Under conditions of short-term plasticity, all five network structures exhibit either subcritical states or Dragon kings, failing to achieve critical states. For network structures with high clustering coefficients, such as regular networks and small world networks, the interplay of short-term and long-term plasticity in our simulations appears to enable the realization of critical states. These modeling results suggest that the effects of network structure and plasticity on neural dynamics are diverse, and that the functional role of plasticity may heavily depend on the current state of neural activity and specific properties of the network. While understanding how synaptic plasticity facilitates the realization and maintenance of critical states is important, its direct relationship with learning and memory formation remains unclear. It is essential to recognize these functions as separate processes.

Previous studies have examined how biologically established synaptic plasticity mechanisms, such as Hebbian learning, are related to the emergence of criticality in neural networks. For example, Uhlig et al. (2013) theoretically analyzed the interaction between Hebbian learning and critical dynamics, showing that neuronal activity near the critical point plays a crucial role in achieving both sparse activity and high memory capacity in associative memory networks. Their study demonstrated that the interplay between Hebbian learning mechanisms and critical dynamics contributes to balancing memory retrieval and stability. These findings suggest that learning-oriented plasticity rules can significantly influence the dynamical regime of neural networks, including the emergence of criticality. In contrast, the synaptic plasticity model employed in the present study is not based on associative learning rules such as Hebbian learning, but rather on a more abstract framework of homeostatic regulation driven by the average activity of neurons. The primary goal of our model is to maintain synaptic strengths near the critical point, thereby allowing the network to self-organize toward a critical state. Consequently, our model is not directly comparable to Hebbian-type rules, which are designed to support learning and memory. Instead, the present study aims to theoretically examine the conditions under which criticality emerges and is stabilized, using a simplified and abstract model. Notably, our additional analysis (see Supplementary Figure S6) revealed that self-organized criticality (SOC) can also be reproduced using an alternative homeostatic plasticity model proposed by Brochini et al. (2016). This result suggests that the emergence of criticality does not depend on a specific plasticity rule. In future work, it will be important to incorporate Hebbian-type rules to further investigate the interaction between criticality and learning functions. However, the primary objective of the present study is to theoretically clarify how homeostatic mechanisms contribute to the maintenance and breakdown of criticality across different network structures. Therefore, our model is not limited to synaptic depression, but instead encompasses a broader class of activity-regulating mechanisms. In this way, our modeling approach provides a complementary perspective to Hebbian-based models and offers novel insights into how network-level homeostasis supports the emergence of critical dynamics in the brain.

The topology of neural networks and the time scale of synaptic plasticity are critical factors in maintaining brain function and adaptability. Understanding how their failure leads to neurological disorders is of paramount importance for unraveling brain pathology. In this study, our modeling results revealed that local connections characteristic of networks such as regular networks and small world networks may facilitate the realization of critical-like states in simplified models when short-term and long-term synaptic plasticity function appropriately. However, for example, when neural networks transition from a small world network to a random network, short-term plasticity (corresponding to the upper-left region in Figure 2 may transform critical-like states into Dragon king dynamics, potentially impacting information processing capacity. On the other hand, while the impact of modularity loss on brain function has been debated (Heiney et al., 2021), our modeling results suggest that even when modularity is lost and networks transition to random structures, critical-like states might remain largely unaffected. However, previous research has suggested that when hierarchical modular structures are assumed, the parameter range of synaptic connection strength that supports critical states widens. This allows neural networks to more easily maintain critical states under varying conditions, demonstrating robust performance against external disturbances (Wang and Zhou, 2012; Moretti and Muñoz, 2013). This may highlight the significant influence of hierarchical structures on maintaining critical-like states. Future research will need to focus on elucidating the specific mechanisms by which changes in modularity and small world properties contribute to brain disorders.

There are several important limitations that should be considered regarding the present model. First, our model does not incorporate inhibitory neurons, which play a central role in regulating neural activity. Inhibitory connections are critical for stabilizing both local and global network dynamics, and it is well established that excitatory-only models tend to promote excessive propagation of neural activity. Previous studies by Shew et al. (2011) has reported that large-scale avalanches resembling dragon king are more likely to emerge without inhibitory mechanisms. Based on these findings, it is highly plausible that the Dragon king phenomena observed in the present study could be mitigated if inhibitory neurons were incorporated into the model. Therefore, the absence of inhibitory regulation may have had a significant impact on the dynamics of the model, potentially introducing behaviors that differ from those seen in actual brain circuits. Second, the networks employed in this study are characterized by exceptionally high clustering coefficients. However, large-scale human brain networks have been reported to exhibit comparatively low clustering coefficients (Achard et al., 2006; Bullmore and Sporns, 2009). Furthermore, a balance between local excitatory and inhibitory connections is maintained in the human brain, and sparse long-range connections enable both global synchrony and efficient information transmission. As a result, brain dynamics exhibit a hybrid structure combining partial modularity with sparse connectivity (Sporns and Zwi, 2004; Kaiser and Hilgetag, 2010). In light of these previous findings, caution is warranted when directly applying the properties of “criticality in highly clustered networks,” as demonstrated in this study, to biological brain networks. In reality, local clustering, inhibitory regulation, and sparse long-range connectivity likely interact intricately, allowing localized firing to be controlled while simultaneously maintaining global efficiency and stability.

Finally, we will discuss future challenges related to this study. One critical question is how critical phenomena support efficient information processing in neural networks. Previous studies have already suggested that critical phenomena are influenced by network structure and synaptic plasticity (Heiney et al., 2021; Plenz et al., 2021), but how these phenomena are involved in the brain's overall information transmission and computation remains insufficiently understood. In particular, many questions remain about the specific mechanisms by which critical phenomena contribute to the brain's adaptive learning capabilities and memory formation. Additionally, understanding how the dragon king observed in this study is related to neurological disorders will be an important research challenge. For instance, the impact of the dragon king on the information processing capacity of neural networks and how it manifests in the early stages of neurological disorders remain unclear. Future research should aim to elucidate the mechanisms through which abnormal neuronal firing leads to various neurological diseases. This understanding will be crucial for advancing our knowledge of brain function and pathology.

## Data Availability

The original contributions presented in the study are included in the article/Supplementary material, further inquiries can be directed to the corresponding author.
