# Supplementary material for: Network structure influences self-organized criticality in neural networks with dynamical synapses
Source: Front Syst Neurosci. 2025 Jun 18;19:1590743. doi: 10.3389/fnsys.2025.1590743 (PMC12213684; doi:10.3389/fnsys.2025.1590743)
Supplement: Supplementary file 1 [file Data_Sheet_1.pdf]

frontiers

Supplementary Material

1 METHODS

1.1 Construction of Modular Networks

The Stochastic Block (SB) model constructs a modular network by generating probabilistically synaptic connections (edges) between neurons (nodes) within and between modules. Therefore, depending on the edge generation probability, the number of synaptic connections can vary significantly. To compare with other networks, such as regular networks, it is necessary to first determine the number of neurons and the expected number of synaptic connections and then derive the edge generation probability to construct the modular network. In this study, synaptic connections within each module are generated with probability p_B, and synaptic connections between modules are generated with probability q_B. Let the number of modules be B, the number of neurons is N (where $N \bmod B = 0$), the total number of synaptic connections within all modules be m_p, the total number of synaptic connections between all modules be m_q (with $m_p : m_q = r : 1$), and the total number of synaptic connections in the entire network be m (where $m_p + m_q = m$). Based on this, we derive the synaptic connection probability p_B within each module and the synaptic connection probability q_B between modules.

First, the total number of synaptic connections within all modules m_p and the total number of synaptic connections between all modules m_q can be derived as:

$$m_p = \frac{1}{r+1}m, \quad m_q = \frac{r}{r+1}m. \tag{S1}$$

Next, let Π_p be the number of neuron pairs within modules, and Π_q be the number of neuron pairs between modules:

$$\Pi_p = \binom{B}{2}\binom{N}{B}^2 = \frac{B(B-1)}{2}\binom{N}{B}^2 = \frac{(B-1)N^2}{2B}, \tag{S2}$$

$$\Pi_q = B\binom{\frac{N}{B}}{2} = \frac{N^2}{2B} - \frac{N}{2}. \tag{S3}$$

From this, the synapse generation probability p_B within each module and the synapse generation probability q_B between modules can be derived as:

$$p_B = \frac{m_p}{\Pi_p} = \frac{\frac{1}{r+1}m}{\frac{(B-1)N^2}{2B}} = \frac{2Bm}{(r+1)(B-1)N^2}, \tag{S4}$$

$$q_B = \frac{m_q}{\Pi_q} = \frac{\frac{r}{r+1}m}{\frac{N^2}{2B} - \frac{N}{2}} = \frac{2Brm}{(r+1)\left(N^2 - BN\right)}. \tag{S5}$$

1.2 Clustering Coefficients of the Network Structures Used in This Study

The clustering coefficients of the network structures used in this study are summarized in the table below (Table. S1).

Table S1. Clustering coefficients of the network structures.

Network Structure	Clustering Coefficient
Regular	0.64270
Small world	0.60630
Module	0.00080
Scale free	0.00450
Random	0.00090

1.3 Fitting to a Truncated Power Law

We explain the method for fitting the CCDF based on the total number of neurons firing in cascades and the duration of firing to a truncated power law. A truncated power law is a model suited for data constrained within a specific lower and upper bound. Here, we collectively refer to the data, such as the total number of neurons firing in cascades and the duration of firing, which are subject to fitting a truncated power law, as D. For the observed data $D_1, D_2, \ldots, D_L$, the log-likelihood function is defined by the following Eq. (S6) (Kagan, 2002):

$$\mathcal{L}(e) = \sum_{l=1}^{L} \log \left(\frac{D_{\max}^{e} D_{\min}^{e}}{D_{\max}^{e} - D_{\min}^{e}} \times D_l^{-1-e} \right) \tag{S6}$$

In this equation, $D_{\min}$ represents the minimum value of the data, and $D_{\max}$ represents the maximum value of the data. The parameter e is the power law exponent estimated from the data and is a critical parameter that determines the shape of the distribution. In the method of maximum likelihood estimation, the value of e that maximizes the log-likelihood function $\mathcal{L}(e)$ is sought. Specifically, by varying e and calculating the value of the likelihood function for the observed data $D_1, D_2, \ldots, D_L$, the value of e that maximizes the likelihood is estimated. This process determines the shape parameter e of the truncated power law that best fits the data. Using this method, we can estimate an appropriate distribution model for data such as the total number of neurons firing in cascades and the firing duration.

1.4 Calculation Method of the Average Firing Rate of Neurons

In this study, the average firing rate ρ of neurons is calculated through a two-step process. First, the instantaneous firing rate $\rho[t]$ at each time point t is defined as the fraction of neurons firing at that time:

$$\rho[t] \equiv \frac{1}{N} \sum_{i=1}^{N} X_i[t]. \tag{S7}$$

$X_i[t]$ represents the firing state (0 or 1) of the neuron i at time t, and N is the total number of neurons. Then, the average firing rate ρ over a time window from t_t to t_f is defined as:

$$\rho \equiv \frac{1}{t_f - t_t + 1} \sum_{t=t_t}^{t_f} \rho[t], \tag{S8}$$

In this study, we set $t_t = 10^4$ and $t_f = 10^5$.

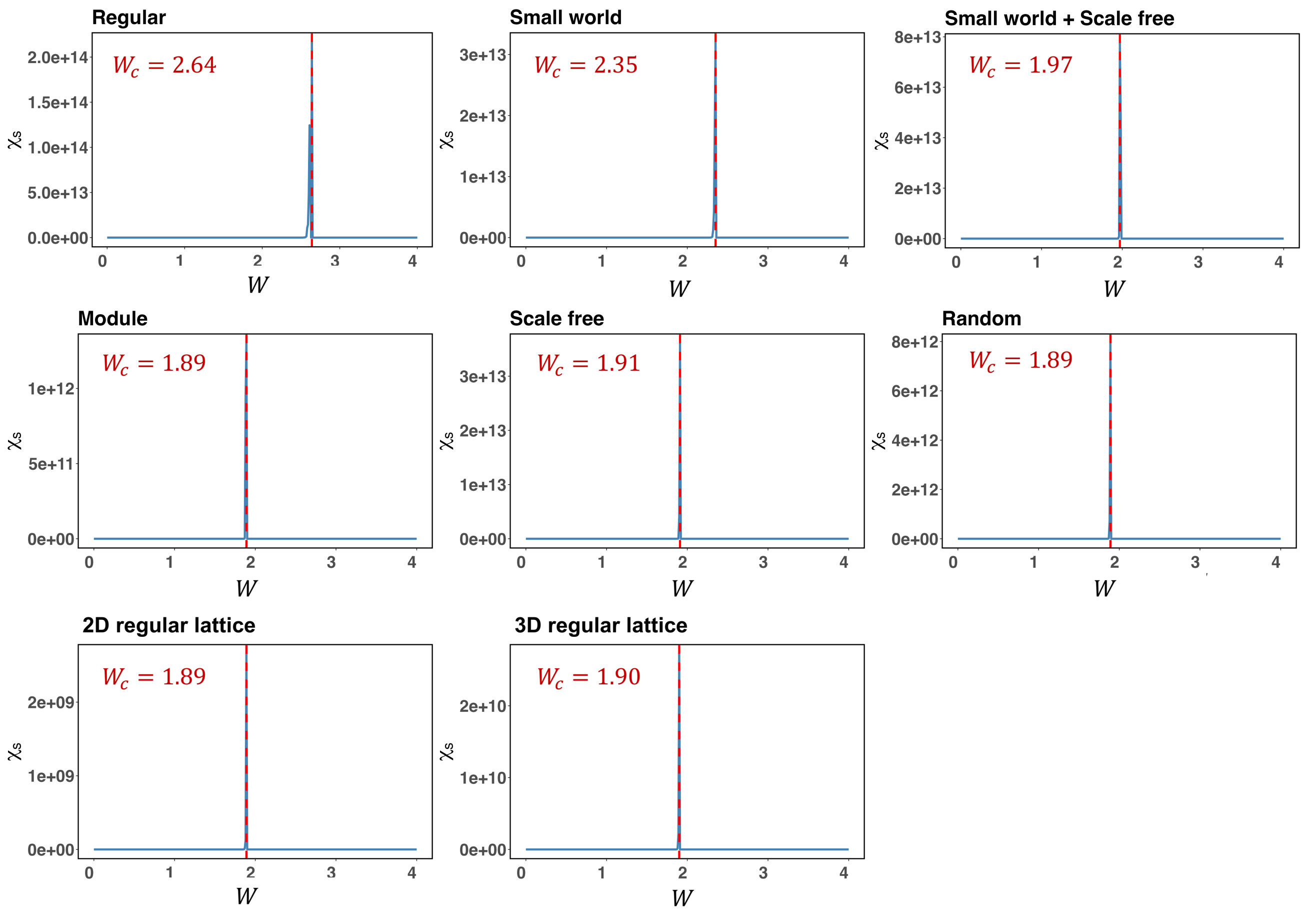

Figure S1: **Avalanche size susceptibility χ_s across different network structures.** This figure shows each network structure's avalanche size susceptibility χ_s. The horizontal axis represents the average synaptic strength W, and the vertical axis represents the susceptibility χ_s. The susceptibility χ_s was calculated according to Eq. (4).

2 RESULTS

2.1 Critical Values for Each Network Structure

The critical synaptic strength W_c estimated for each network structure is shown in Fig. S1. Using the estimated critical value as the initial condition, we simulated the time evolution of synaptic strength. The results are presented in Fig. 1 & 2.

2.2 Classification Results of Neural Network States

The conditions of synaptic plasticity time scales under which the neural network state reaches one of "Dragon king," "Subcritical," "Critical," or "Supercritical" are shown (Fig. S2). A state is defined as "Dragon king" when the firing distribution deviates above the truncated power law (Fig. S2A). When the maximum element of the vector s is less than or equal to $10^{2.5}$, the state is classified as "Subcritical," as an insufficient number of neurons fired in a cascade (Fig. S2B). In the case of "Supercritical," there is only one data point plotted, as no instance of neuron inactivity occurred, resulting in a large-scale cascade of neuron firing (Fig. S2D).

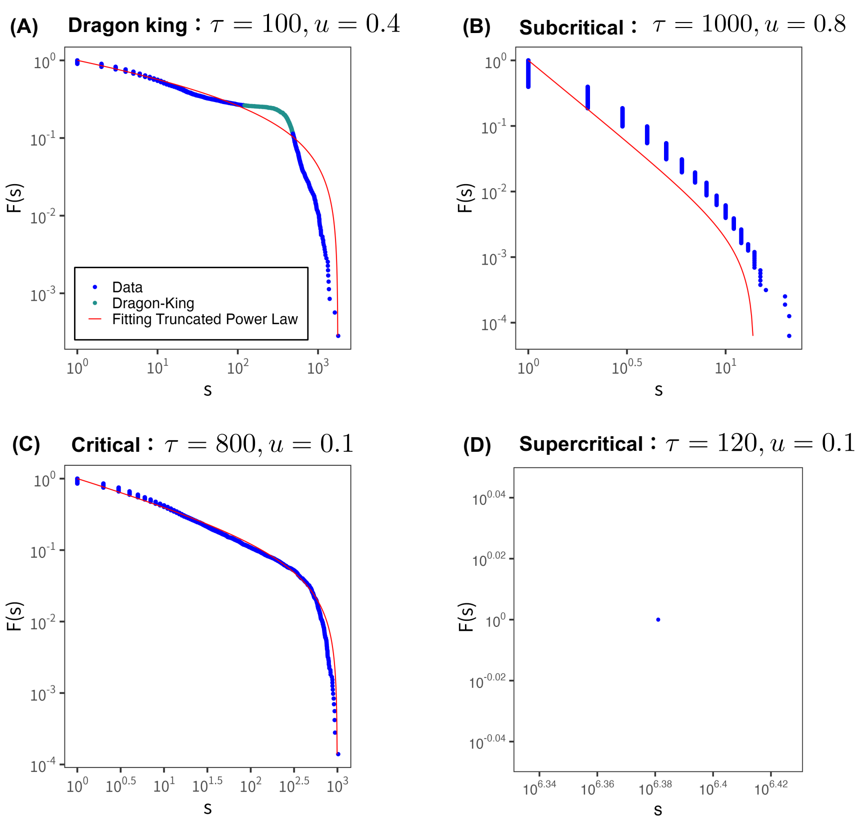

Figure S2: **Classification results of neural network states.** The figure shows the classification of the neuron firing distribution obtained from the numerical analysis of the synaptic plasticity model in Eq. (5) into "Dragon king," "Subcritical," "Critical," and "Supercritical." Blue dots represent data points, the red curve shows the fitting result from the truncated power law, and green dots represent data points classified as Dragon king. All results are from random networks. **(A)** Dragon king: $\tau = 100, u = 0.4$. **(B)** Subcritical: $\tau = 1000, u = 0.8$. **(C)** Critical: $\tau = 800, u = 0.1$. **(D)** Supercritical: $\tau = 120, u = 0.1$.

2.3 Probability Density Function of Neuronal Avalanche Size

As a supplementary analysis to Fig. 1B, the probability density function (PDF) corresponding to the complementary cumulative distribution function (CCDF) of neuronal firing frequency is shown in Fig. S3.

2.4 On the Scaling Laws Between Multiple Power Laws

Near the critical value W_c, multiple power-law distributions can emerge, not only for the number of neurons firing in cascades but also for the duration of the firing events and the relationship between these two. A relationship known as the "scaling law" is expected to hold among these different power exponents under specific theoretical assumptions. Let α be the exponent for the number of neurons firing in cascades (dependent variable), and β be the exponent for the duration of firing events (dependent variable). Let γ represent the slope characterizing the relationship between these two variables. In an idealized critical state, the following relation is predicted: $(\alpha - 1)/(\beta - 1) = \gamma$. Numerical analysis using a mathematical model that incorporates synaptic plasticity across various network structures (Fig. S10) showed that the data points for cascade size and duration approximately follow this scaling relation in all networks. However, deviations from the theoretical slope were also observed. This may reflect the fact that certain assumptions required for scaling laws—such as temporal scale separation and stationarity—are not strictly satisfied in

self-organized neural systems. Therefore, while the results suggest consistency with critical-like behavior, they should not be interpreted as definitive evidence that the scaling law strictly holds or that the system is in a precise critical state.

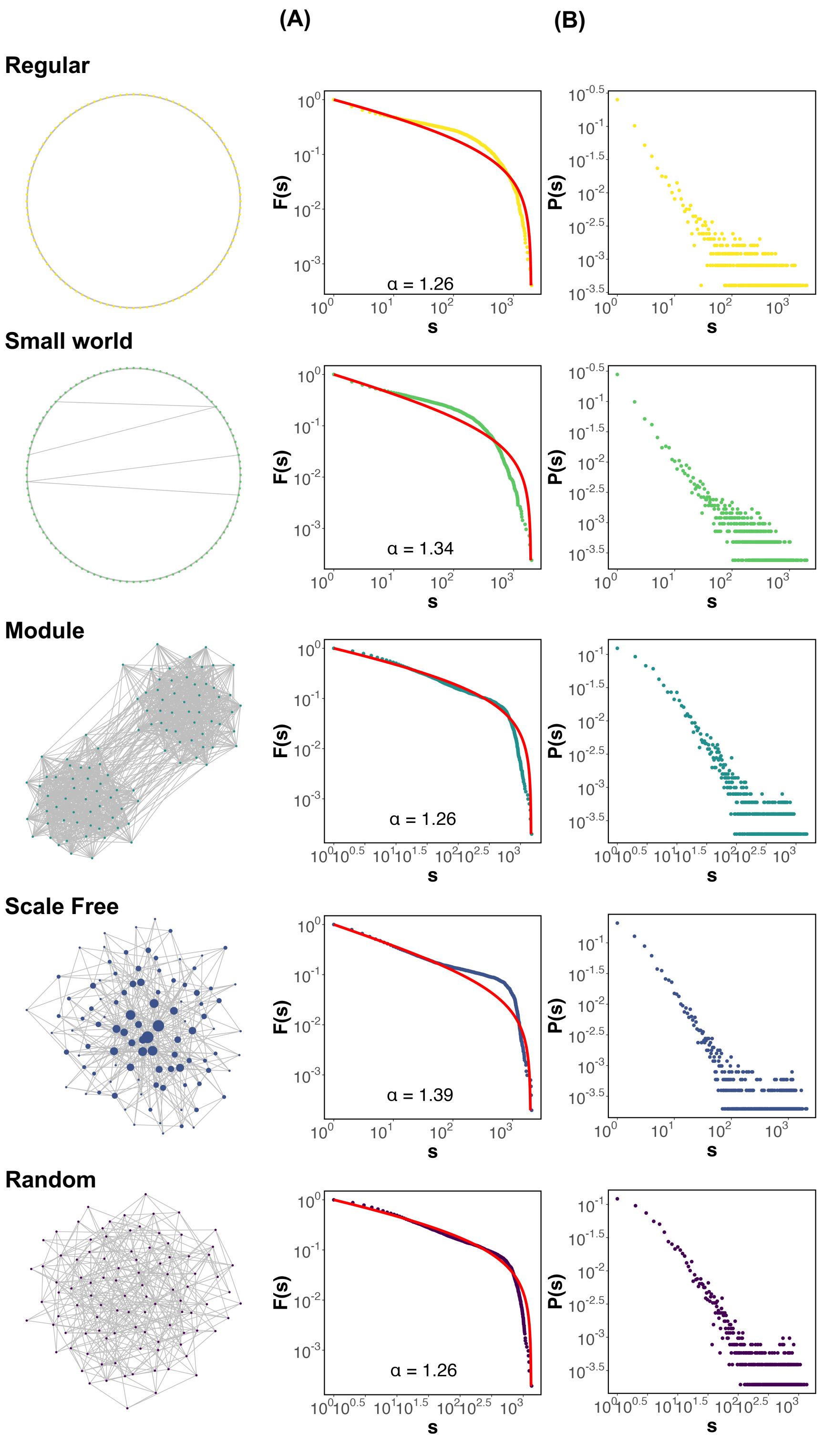

Figure S3: **Comparison of neuronal firing frequency distributions across different visualizations.** The numerical simulation was conducted under the same conditions as in Fig. 1. **(A)** The neuronal firing frequency distribution is shown as a complementary cumulative distribution function (CCDF). The red solid line represents a truncated power-law distribution fitted to the data. This figure reproduces the results shown in Fig. 1B. **(B)** The probability density function (PDF) corresponding to the firing frequency distribution in (A) is presented.

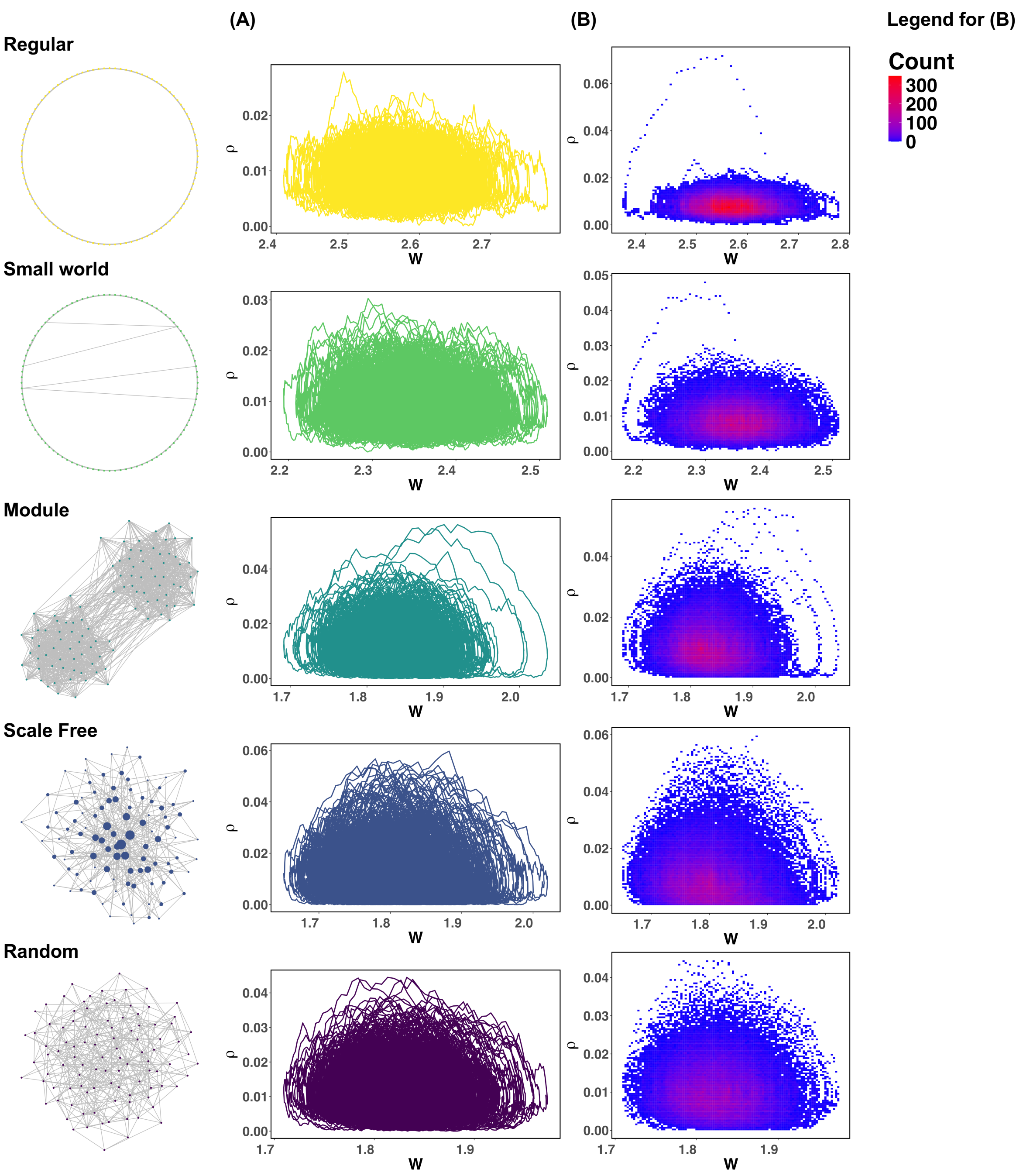

Figure S4: **The relationship between the average synaptic strength W and the average firing rate ρ.** The conditions for the numerical simulation experiment are the same as in Fig. 1. **(A)** The trajectory of the average synaptic strength W and the average firing rate ρ during $t = 10^4 \sim 10^5$. **(B)** Image plot of the average synaptic strength W and the average firing rate ρ over the entire time.

2.5 The Relationship Between Average Synaptic Strength and Average Firing Rate

The relationship between the average synaptic strength W and the average firing frequency ρ under the numerical simulation conditions of Fig. 1 is shown. According to Eq. (5), the more neurons fire, the more the average synaptic strength W decreases, resulting in trajectories like those shown in Fig. S4. It can be

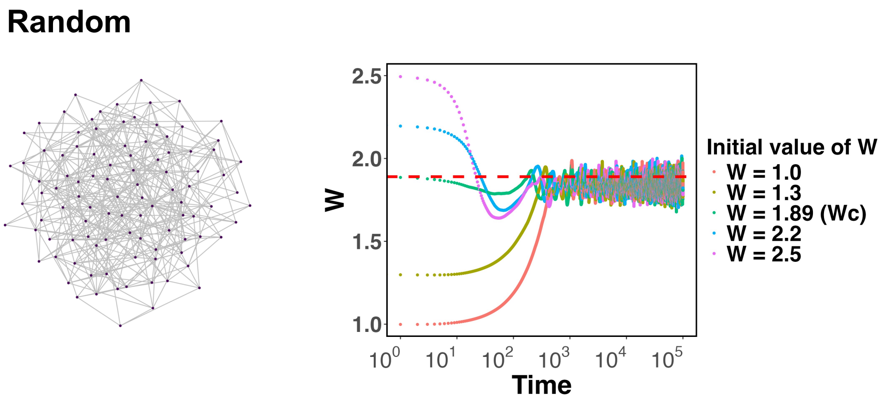

Figure S5: **Time evolution of the average synaptic strength W in a Random network from different initial values.** This figure shows the results of simulations starting from five different initial values of the average synaptic strength W in a Random network. The numerical analysis conditions are identical to those used for Random network in Fig. 1.

observed that in modular networks, scale free networks, and random networks, the behavior is near the critical value W_c.

2.6 Initial Setting of the Average Synaptic Strength and Validation of Convergence

In this study, the initial value of the average synaptic strength W in the simulations was set to the critical value W_c. This choice was made with the expectation that neural dynamics would quickly approach critical behavior from the initial state. However, it is known that even when the initial value is set to a different level, the dynamics of synaptic plasticity eventually leads the system to converge to W_c. This behavior has also been reported in previous studies (Pazzini et al., 2021). In this study, we also performed simulations starting from multiple different initial values of the average synaptic strength W in Random networks, and the results are shown below (Fig. S5). Regardless of the initial conditions, in all cases the system ultimately converged to W_c. These findings indicate that differences in the initial value do not affect the main conclusions of this study.

2.7 Simulations under Different Synaptic Plasticity Rules

In addition to the synaptic plasticity model used in the main analysis, we implemented an alternative plasticity rule proposed in a previous study and conducted numerical simulations under the same conditions. The formulation of this model is as follows:

$$W_{ij}[t+1] = W_{ij}[t] + \frac{1}{\tau}(A - W_{ij}[t]) - uW_{ij}[t]X_j[t]. \tag{S9}$$

Eq. (S9) is based on the model proposed by Brochini et al. (2016). As in the model used in our main analysis, the average synaptic strength W remained near the critical value W_c, and the distribution of neuronal firing frequencies followed a truncated power law (Fig. S6). These results demonstrate that self-organized criticality (SOC) can be robustly achieved not only with our original model but also with alternative plasticity rules. In particular, the homeostatic regulation toward a target value A appears to play a critical role in maintaining the system near criticality.

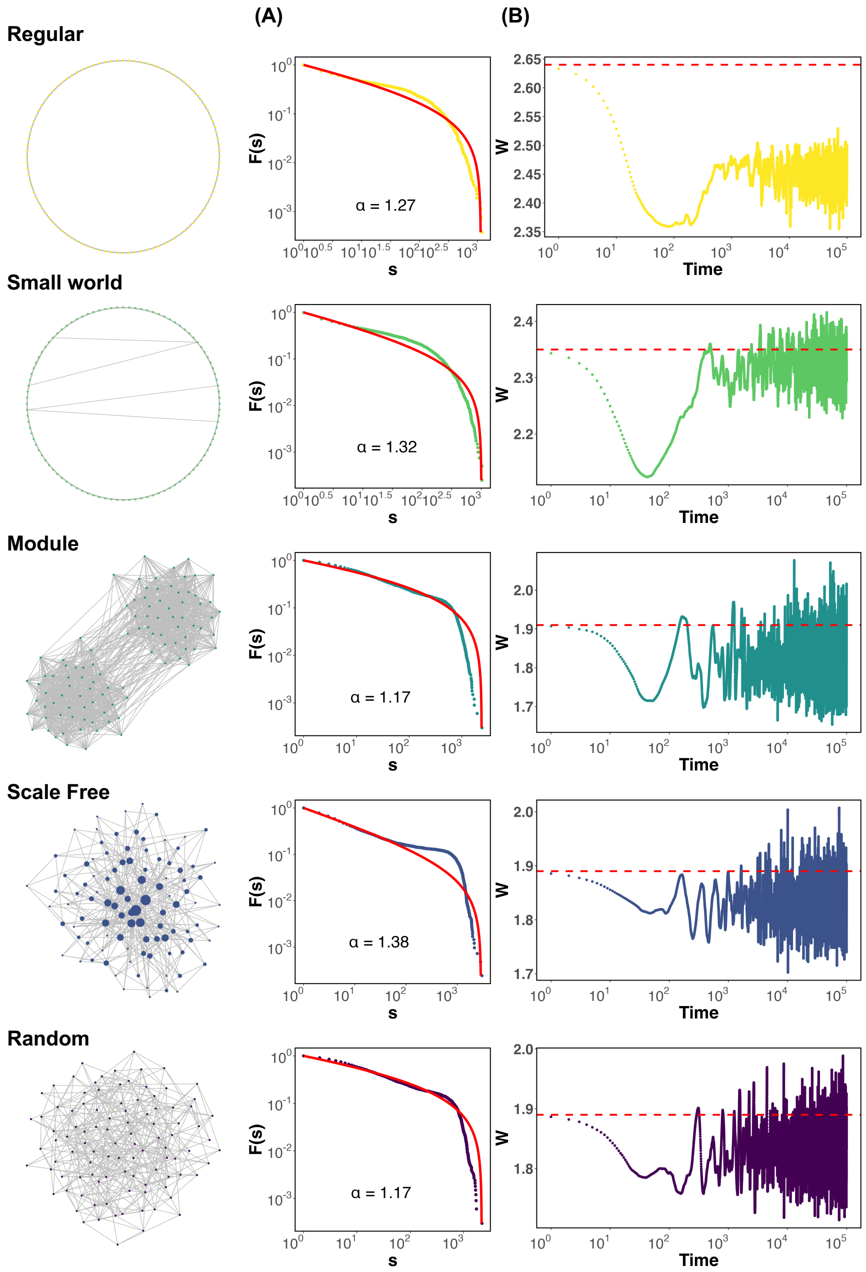

Figure S6: **Simulation results based on the synaptic plasticity model defined in Eq. (S9).** Numerical simulations were conducted under the same conditions as in Fig. 1, but the synaptic plasticity model was changed from Eq. 5 to Eq. S9.**(A)** CCDF based on the total number of neurons that have been fired in a chain reaction, s. The red solid line represents the result of fitting the distribution to a truncated power law. α is the power law exponent. **(B)** Visualization of the time evolution of the average synaptic strength W. The red dashed line represents the critical value W_c.

2.8 Correlation Between the Maintenance of Criticality and the Deviation of the Mean Synaptic Strength from Its Critical Value

To examine the correlation between the maintenance of criticality and the deviation of the mean synaptic strength from its critical value, we created box plots for each network structure, illustrating the distribution

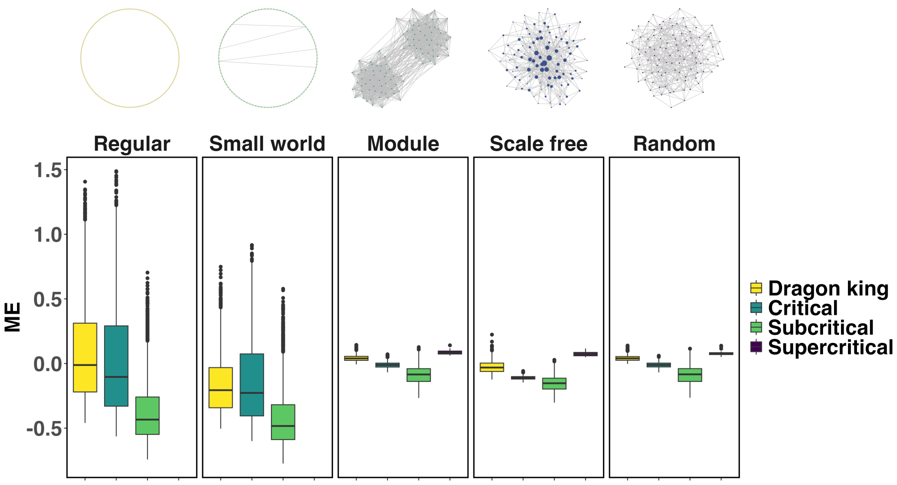

Figure S7: **Correlation Between the Maintenance of Criticality and the Deviation of the Mean Synaptic Strength from Its Critical Value.** A box plot of ME for each synaptic plasticity time scale, categorized into Dragon king, critical, subcritical, and supercritical states, was drawn for each network structure. In the Regular and Small world networks, no box plot is shown for the supercritical state because no synaptic plasticity time scales were identified as being in a supercritical state. For the relationship between the maintenance of criticality and the deviation of the mean synaptic strength from its critical value, refer to Fig. 2.

of ME across different time scales of synaptic plasticity classified into Dragon king, Critical, Subcritical, and Supercritical states (Fig. S7). The results revealed that in Regular and Small world networks, the mean ME in the subcritical state was lower compared to other states. In contrast, in other network structures, ME values remained consistent regardless of whether the critical state was maintained. These findings suggest that the correlation between the maintenance of criticality and the deviation of the mean synaptic strength from its critical value depends on the network structure.

2.9 Validation in Networks with Both Hub Structures and High Clustering Coefficients

In this study, we demonstrated that in network structures with high clustering coefficients, such as Regular and Small world networks, the average synaptic strength W deviates from the critical value W_c, whereas in networks with hub structures, such as Scale-free networks, Dragon king events—characterized by deviations from the power law distribution of neuronal firing frequencies—emerge. To investigate whether both phenomena could occur simultaneously in networks exhibiting both high clustering and hub structures, we constructed networks using the Holme-Kim model(Holme and Kim, 2002) and conducted similar simulations. The Holme-Kim model extends the classical Barabási–Albert (BA) model(Barabási and Albert, 1999) by incorporating mechanisms that enable the generation of networks with both scale-free degree distributions and high clustering coefficients. In the BA model, networks grow through preferential attachment, whereby newly added nodes are more likely to connect to high-degree nodes. However, this process alone fails to reproduce the high clustering observed in many real-world networks, such as social or biological systems. Holme and Kim (2002) proposed a simple yet effective modification: after a new node connects via preferential attachment, it forms a triangle (triadic closure) with the neighbors of the connected node with a certain probability p. This process enhances the local clustering within the network. The clustering coefficient of the network constructed using the Holme-Kim model in our simulations was 0.5005. The results of the simulations using the Holme-Kim network are shown in Fig. S8. We found

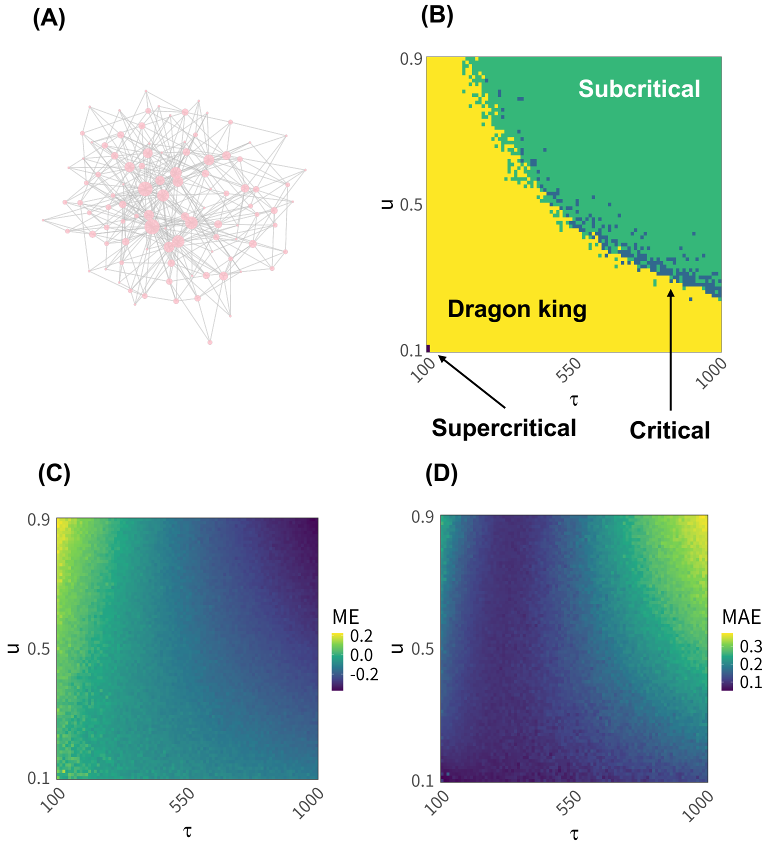

Figure S8: **Characteristics of neural activity in networks with hub structures and high clustering coefficients.** Simulation results using networks constructed with the Holme-Kim model, under various combinations of synaptic plasticity parameters τ and u. **(A)** Visualization of the network structure. For simplicity, a network with $N = 100$ nodes and $m = 400$ edges is used. **(B)** Numerical analysis of whether a critical state is achieved under various synaptic plasticity conditions. **(C)** Deviation of the average synaptic strength W from the critical value W_c, calculated as the ME indicator based on Eq. (6) and visualized. **(D)** Deviation of the average synaptic strength W from the critical value W_c, calculated as the MAE indicator based on Eq. (7) and visualized.

that Dragon king events emerged across most combinations of the synaptic plasticity parameters τ and u (Fig. S8B). Additionally, under certain combinations of τ and u, the average synaptic strength W deviated from the critical value W_c (Fig. S8C, D). These findings indicate that both the emergence of Dragon king events associated with hub structures and the deviation of W due to high clustering can coexist within a single network. In general, these results reinforce the conclusions of this study: namely, that a high clustering coefficient leads to the deviation of the average synaptic strength W from the critical value W_c. At the same time, the presence of hub structures induces the emergence of Dragon king.

2.10 Structural Variations and Critical Dynamics in Regular and Small World Networks

We conducted numerical simulations using Watts-Strogatz (WS) networks with rewiring probabilities $p = 0.0001, p = 0.001$, and $p = 0.1$, in addition to the previously analyzed cases of $p = 0.0$ and $p = 0.01$. This allowed us to investigate a broader range of structural diversity from regular to small world networks. All WS networks were constructed based on a one-dimensional ring lattice, consistent with the schematic presented in the main text. Across all network structures, the neuronal firing frequency distributions in

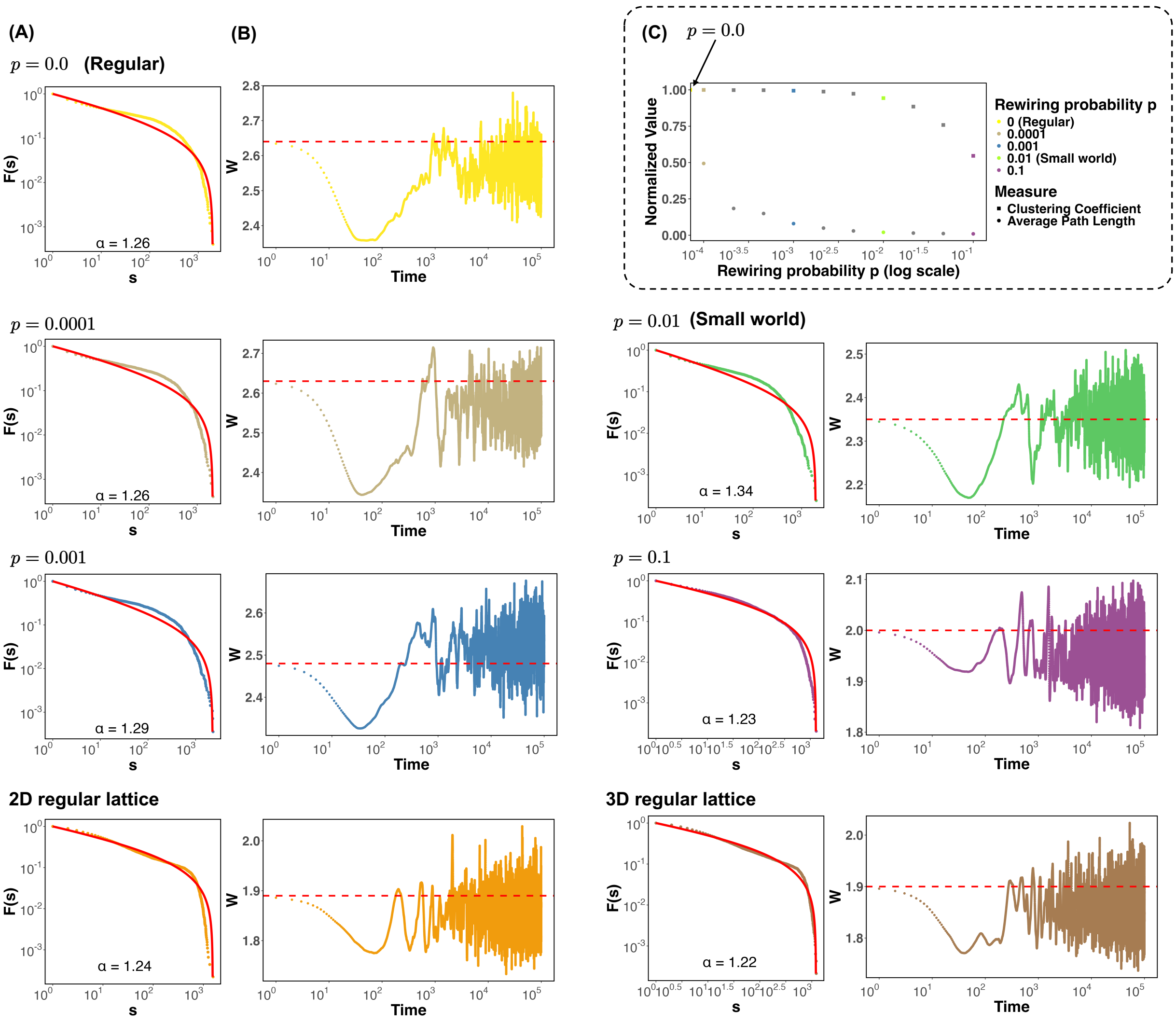

Figure S9: **Numerical analysis of Watts–Strogatz (WS) networks with varying rewiring probabilities, 2D lattice networks, and 3D lattice networks.** We conducted numerical simulations using Watts–Strogatz (WS) networks with various rewiring probabilities p, along with 2D and 3D regular lattice networks, thereby covering a range of network structures. The simulation conditions were identical to those used in Fig. 1. **(A)** Complementary cumulative distribution functions (CCDFs) of neuronal firing frequencies. The red solid line represents a truncated power law distribution fitted to the empirical data. **(B)** The temporal evolution of the average synaptic strength W illustrates its behavior around the critical value W_c. The red dashed line indicates the critical threshold W_c. **(C)** Changes in the clustering coefficient and characteristic path length as a function of the rewiring probability p (x-axis). Both values are normalized. This panel was adapted from Watts and Strogatz (1998).

networks with $p = 0.0$ to $p = 0.01$ -corresponding to regular and small world topologies- deviated from a power-law distribution. In contrast, the distribution in the random network with $p = 0.1$ followed a power-law behavior (Fig. S9A). Moreover, regardless of the rewiring probability p, simulations under the synaptic plasticity parameters $\tau = 500$ and $u = 0.1$ showed that the average synaptic strength W remained near the critical value W_c (Fig. S9B). These results suggest that a critical state can emerge under fixed plasticity parameters irrespective of network structure, thereby providing evidence for the potential structural independence of self-organized criticality. Additionally, Fig. S9C presents the changes in

clustering coefficient and characteristic path length as a function of the rewiring probability p, quantitatively confirming that the networks used in this study exhibit small world properties. In addition, we constructed two-dimensional and three-dimensional lattice networks with the same number of nodes and average degree as the one-dimensional regular network to evaluate the influence of spatial dimensionality. Simulations conducted under the same synaptic plasticity parameters revealed that, unlike in the one-dimensional regular network, the average synaptic strength W in both the 2D and 3D lattice networks remained close to the critical value W_c.

2.11 The State of Neural Networks When Varying the Leak Parameter in the LIF Model

The effect of changing the leak parameter μ in the LIF model on the state of the neural network is shown (Fig. S11). As a result, it was revealed that the structure of the neural network changes significantly depending on the value of the leak parameter μ.

2.12 The State of Neural Networks When Varying the Average Degree

The effect of changing the average degree in the neural network on its state is shown (Fig. S12). As a result, it was revealed that, except for networks with high clustering coefficients, such as regular and small world networks, the state of the neural network shows little change as the average degree is varied.

2.13 The State of Neural Networks When Varying the Number of Modules

This figure illustrates how the state of the neural network changes when varying the number of modules B in a modular network (Fig. S13). The results show that the state of the neural network exhibits little change depending on the number of modules B.

2.14 Robustness of Dragon King Classification to Threshold Criteria

In this study, events were classified as Dragon kings when the firing frequency of neurons, represented by the complementary cumulative distribution function (CCDF), deviated by more than 1.1 times from the cumulative frequency expected under a truncated power law distribution (Subsect. 2.6). The results obtained when the classification threshold was modified to 1.3 or 1.5 times are shown in Fig. S14. These changes in the threshold did not lead to any substantial differences in the results.

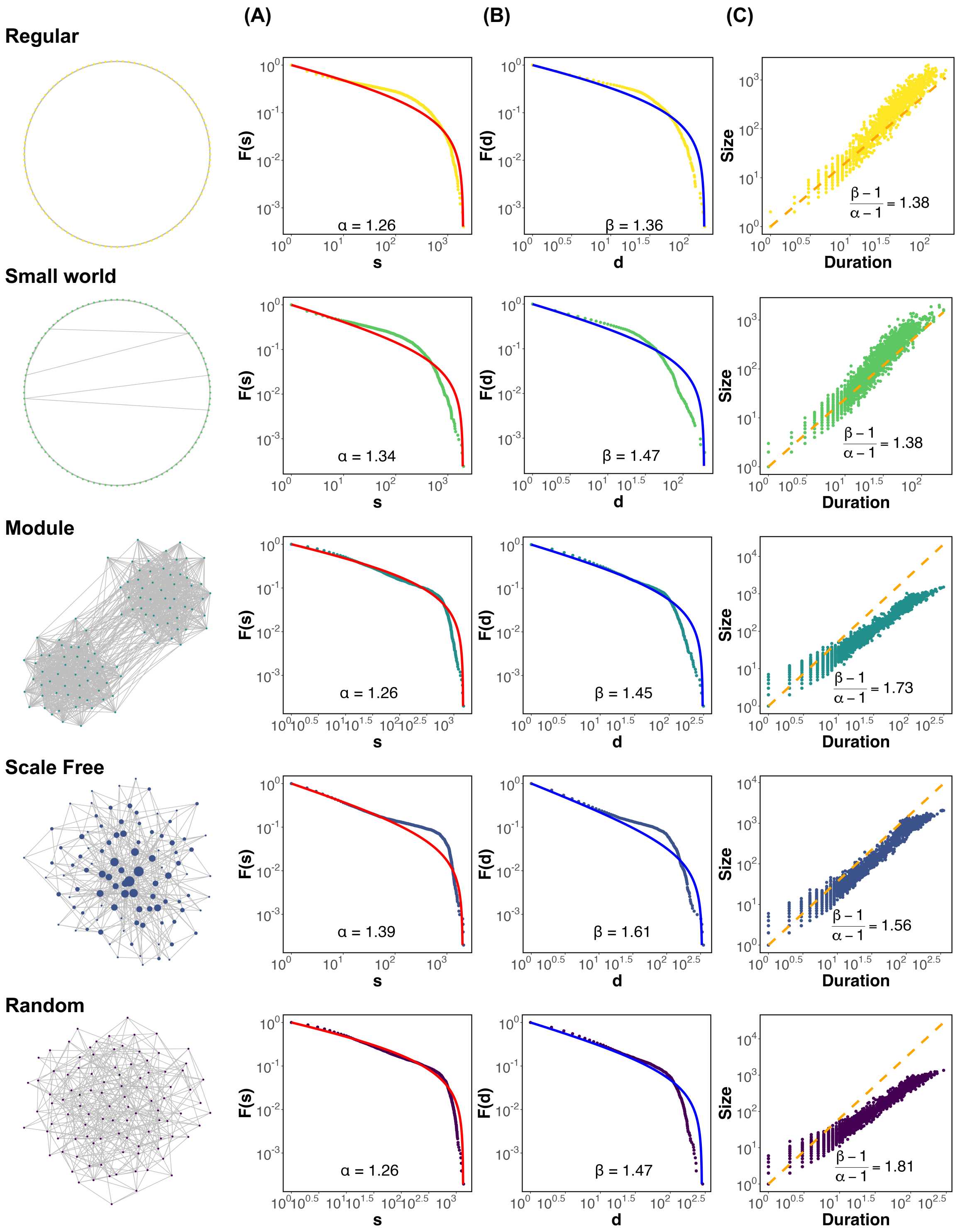

Figure S10: **The number of neurons firing in cascades the duration of firing, and the relationship between these two.** The scaling laws between multiple power laws are verified by plotting the CCDF for the number of neurons firing in cascades, the CCDF for the duration of firing, and a scatter plot showing the relationship between these two for each network structure. **(A)** CCDF based on the total number of neurons firing in cascades. This is the same as Fig. 1B. **(B)** CCDF based on the duration of cascaded firing. d represents the duration of cascaded firing. The blue solid line shows the fitting result of the truncated power law. β is the power law exponent. **(C)** Scatter plot of the number of neurons firing in cascades and the duration of firing. The orange dashed line shows the slope $(\alpha – 1)/(\beta – 1) = \gamma$.

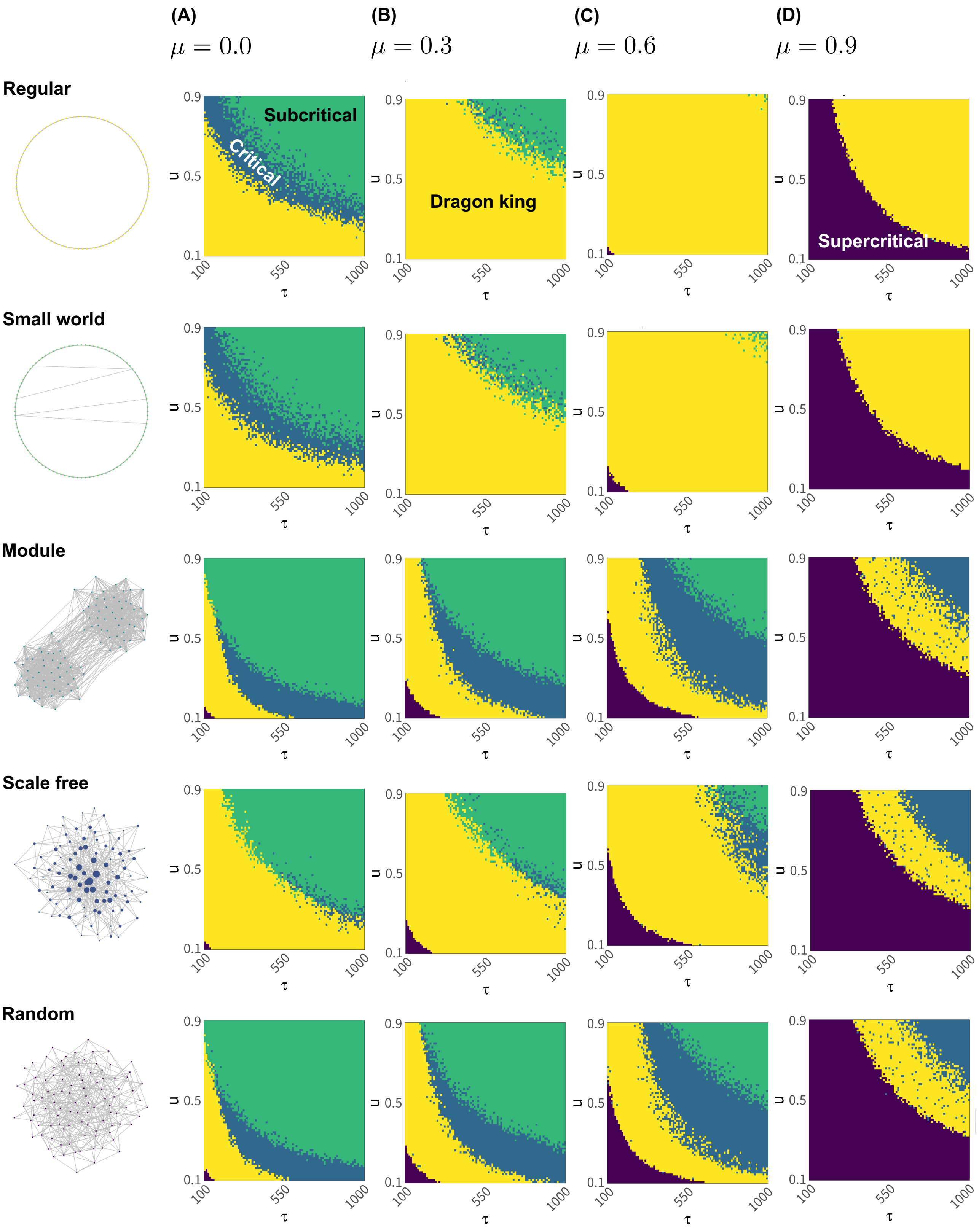

Figure S11: **The state of the neural network when varying the leak parameter in the LIF model.** Assuming synaptic plasticity within the range of $100 \leq \tau \leq 1000$ and $0.1 \leq u \leq 0.9$ in Eq. (5), numerical analysis was conducted to show under which synaptic plasticity conditions the critical state is realized, for each value of the leak parameter μ in the LIF model and for each network structure. The results for $\mu = 0.0$ are the same as Fig. 2A. **(A)** $\mu = 0.0$. **(B)** $\mu = 0.3$. **(C)** $\mu = 0.6$. **(D)** $\mu = 0.9$.

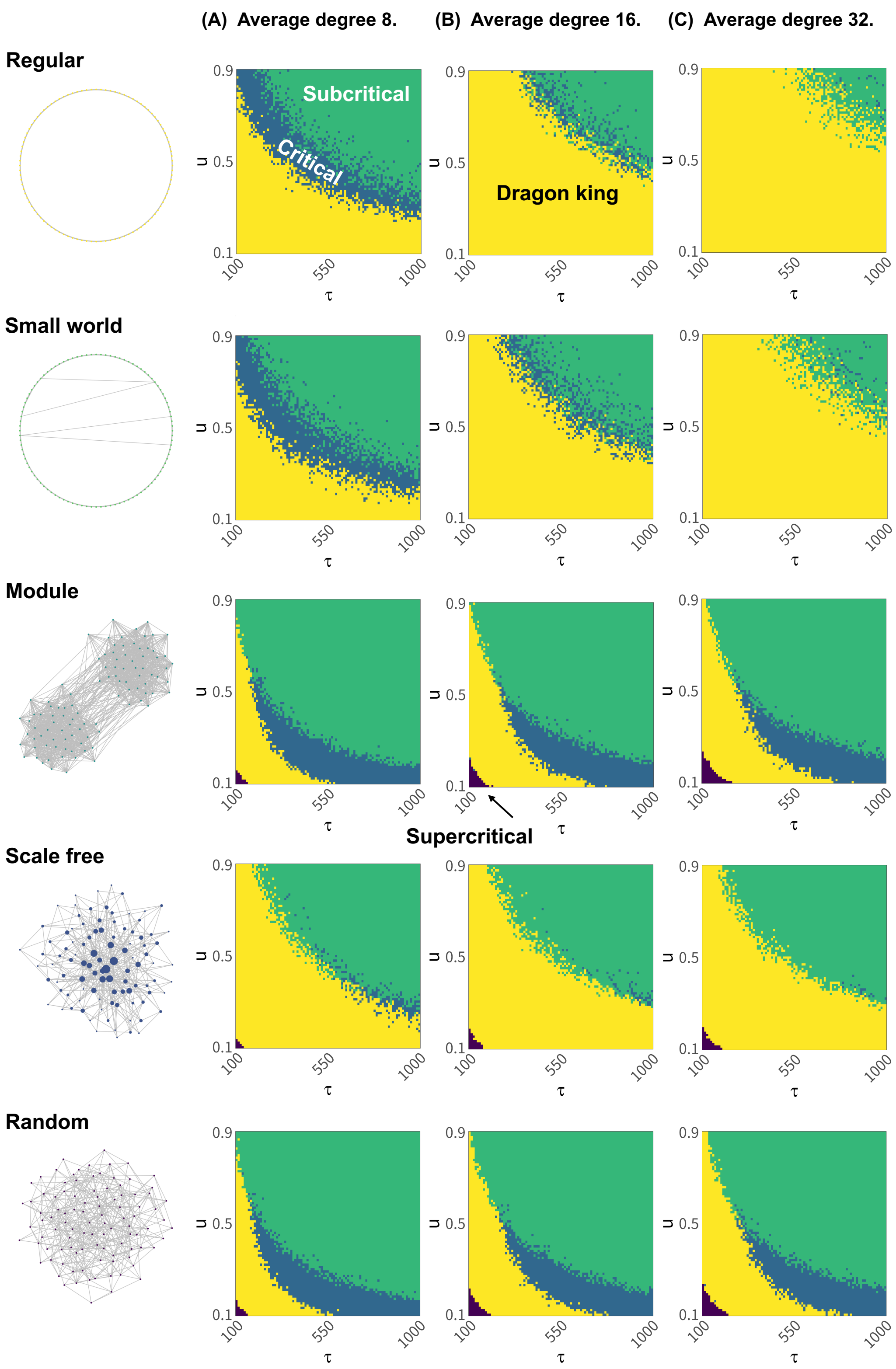

Figure S12: **The state of the neural network when varying the average degree in the neural network.** Assuming synaptic plasticity within the range of $100 \leq \tau \leq 1000$ and $0.1 \leq u \leq 0.9$ in Eq. (5), numerical analysis was conducted to show under which synaptic plasticity conditions the critical state is realized for neural networks with different average degrees. The results for an average degree of 8 are the same as Fig. 2A. **(A)** Average degree 8. **(B)** Average degree 16. **(C)** Average degree 32.

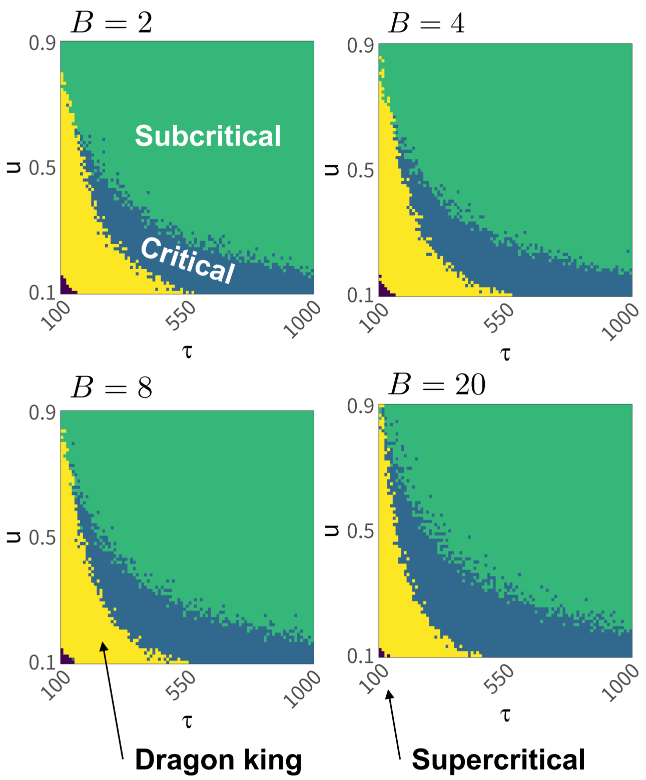

Figure S13: **The neural network state when varying the number of modules B in the neural network.** Assuming synaptic plasticity within the range of $100 \leq \tau \leq 1000$ and $0.1 \leq u \leq 0.9$ in Eq. (5), numerical analysis was conducted to show under which synaptic plasticity conditions the critical state is realized for neural networks with different numbers of modules B. The results for $B = 2$ are the same as Fig. 2A (Module). **(A)** $B = 2$. **(B)** $B = 4$. **(C)** $B = 8$. **(D)** $B = 20$.

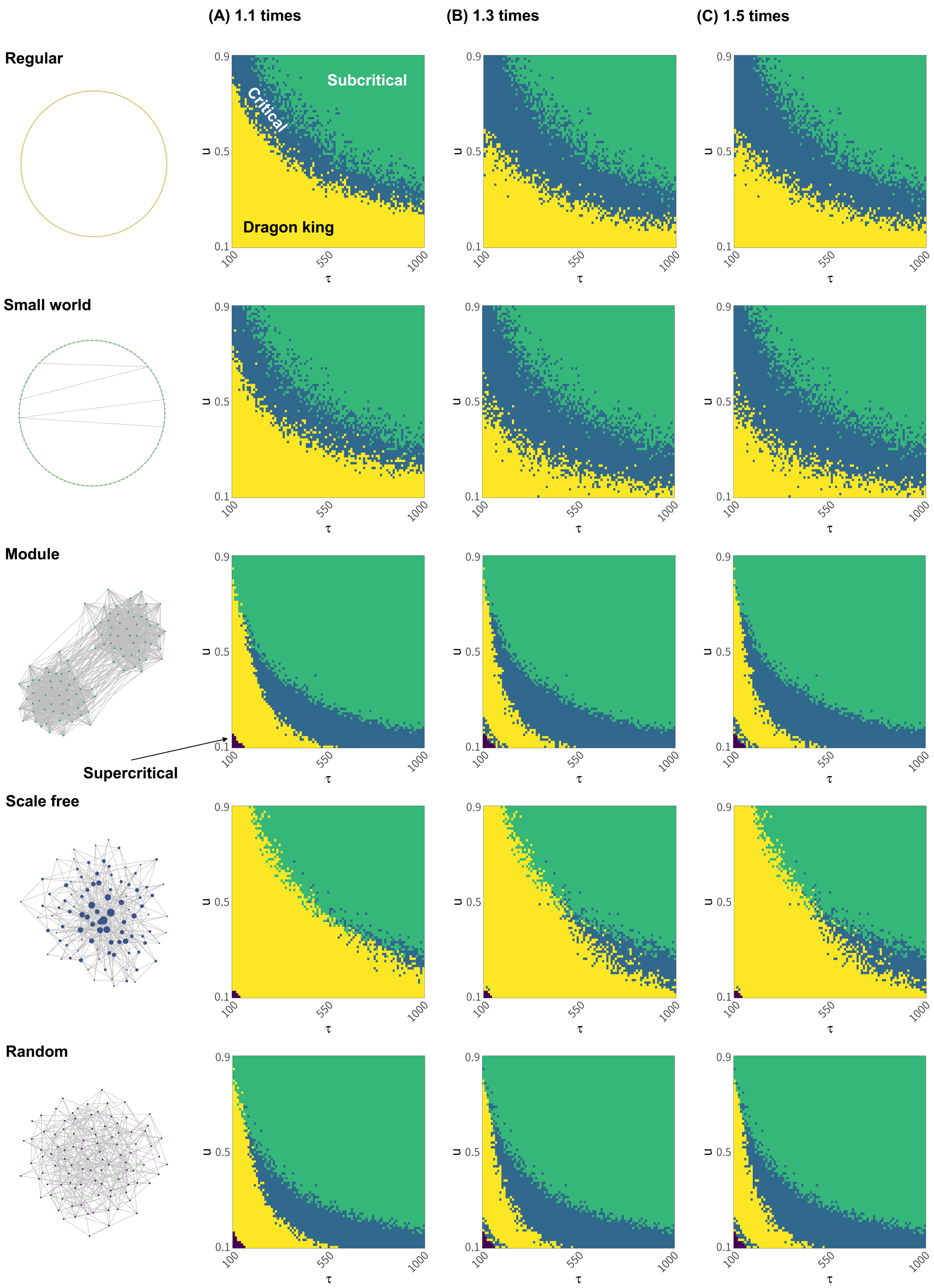

Figure S14: **Comparison of Dragon king classification under different threshold criteria.** This figure shows the results when events are classified as Dragon kings based on whether the neuronal firing frequency (CCDF) deviates by more than 1.1, 1.3, or 1.5 times from the cumulative frequency expected under a truncated power law distribution. Panel (A) corresponds to the same result as shown in Fig. 2B. (A) 1.1× threshold, (B) 1.3× threshold, (C) 1.5× threshold.

REFERENCES

Barabási, A.-L. and Albert, R. (1999). Emergence of scaling in random networks. *science* 286, 509–512

Brochini, L., de Andrade Costa, A., Abadi, M., Roque, A. C., Stolfi, J., and Kinouchi, O. (2016). Phase transitions and self-organized criticality in networks of stochastic spiking neurons. *Scientific reports* 6, 35831

Holme, P. and Kim, B. J. (2002). Growing scale-free networks with tunable clustering. *Physical review E* 65, 026107

Kagan, Y. Y. (2002). Seismic moment distribution revisited: I. statistical results. *Geophysical Journal International* 148, 520–541

Pazzini, R., Kinouchi, O., and Costa, A. A. (2021). Neuronal avalanches in watts-strogatz networks of stochastic spiking neurons. *Physical Review E* 104, 014137

Watts, D. J. and Strogatz, S. H. (1998). Collective dynamics of 'small-world'networks. *nature* 393, 440–442
